# Response-Related Signals Increase Confidence But Not Metacognitive Performance

**DOI:** 10.1523/ENEURO.0326-19.2020

**Published:** 2020-05-20

**Authors:** Elisa Filevich, Christina Koß, Nathan Faivre

**Affiliations:** 1 Bernstein Center for Computational Neuroscience Berlin, 10115 Berlin, Germany; 2 Research Training Group 2386 “Extrospection”, Humboldt-Universität zu Berlin, 10099 Berlin, Germany; 3 Institute of Psychology, Humboldt-Universität zu Berlin, 10099 Berlin, Germany; 4 Laboratory of Cognitive Neuroscience, Brain Mind Institute, Faculty of Life Sciences, Swiss Federal Institute of Technology, 8092 Geneva, Switzerland; 5 Center for Neuroprosthetics, Faculty of Life Sciences, Swiss Federal Institute of Technology, 8092 Geneva, Switzerland; 6 Laboratoire de Psychologie et Neurocognition, CNRS UMR 5105, Université Grenoble Alpes, 38400 Saint-Martin-d'Hères, France

**Keywords:** confidence, metacognition

## Abstract

Confidence judgments are a central tool in metacognition research. In a typical task, participants first perform perceptual (first-order) decisions and then rate their confidence in these decisions. The relationship between confidence and first-order accuracy is taken as a measure of metacognitive performance. Confidence is often assumed to stem from decision-monitoring processes alone, but processes that co-occur with the first-order decision may also play a role in confidence formation. In fact, some recent studies have revealed that directly manipulating motor regions in the brain, or the time of first-order decisions relative to second-order decisions, affects confidence judgments. This finding suggests that confidence could be informed by a readout of reaction times in addition to decision-monitoring processes. To test this possibility, we assessed the contribution of response-related signals to confidence and, in particular, to metacognitive performance (i.e., a measure of the adequacy of these confidence judgments). In human volunteers, we measured the effect of making an overt (vs covert) decision, as well as the effect of pairing an action to the stimulus about which the first-order decision is made. Against our expectations, we found no differences in overall confidence or metacognitive performance when first-order responses were covert as opposed to overt. Further, actions paired to visual stimuli presented led to higher confidence ratings, but did not affect metacognitive performance. These results suggest that confidence ratings do not always incorporate motor information.

## Abstract (precis)

Confidence judgments are a central tool in metacognition research. In a typical task, participants first perform perceptual (first-order) decisions and then rate their confidence in these decisions. The relationship between confidence and first-order accuracy is taken as a measure of metacognitive performance. Confidence is often assumed to stem from decision-monitoring processes alone, but processes that co-occur with the first-order decision may also play a role in confidence formation.

## Significance Statement

To measure metacognition, or the ability to monitor one’s own thoughts, experimental tasks often require human volunteers to, first, make a perceptual decision (“first-order task”) and, then, rate their confidence in their own decision (“second-order task”). In this paradigm, both first-order and second-order information could, in principle, influence confidence judgments. But only the latter is truly metacognitive. To determine whether confidence is a valid metacognitive measure, we compared confidence ratings between the following two conditions: with overt responses, where participants provided both first-order and second-order responses; and with covert responses where participants reported their confidence in a decision that they had not executed. Removing first-order decisions did not affect confidence, which validates confidence as an introspective measure.

## Introduction

Confidence judgments about one’s own perception have been exploited in recent years as a useful way to probe introspection (Fleming and Dolan, 2012). In a now standard paradigm, participants first make a binary decision (typically, a perceptual or memory judgment, first-order task) and afterward rate the confidence in their response (second-order task). Metacognitive performance is measured as the relationship between accuracy in the first-order task and confidence in the second-order task (Fleming and Lau, 2014). Crucially, it is still unclear what confidence reports actually represent, as the variables participants compute to generate them remain latent.

**Table 1:** Statistical table

	Data structure	Type of test	Power/effect size	Statistic
a: Performance in first-order task (CR+R+ vs CR− R+)	Mean per subject (continuous)	Bayesian *t* test	*d* = −0.02	BF_10_ = 0.22
b: Mean perceptual evidence (CR+R+ vs CR−R+)	Mean per subject (continuous)	Bayesian *t* test	*d* = 0.36	BF_10_ = 0.54
c: interaction between overt first-order (R+/R−) and continuous report (CR+/CR−) on mean confidence (all trials)	Single-trial confidence ratings (continuous)	Bayesian mixed-effects linear regression	Mean = −0.02 ± 0.01	Evidence ratio = 0.10
d: Main effect of CR+ on confidence (all trials)	Single-trial confidence ratings (continuous)	Bayesian mixed-effects linear regression	Mean = 0.04 ± 0.02	Evidence ratio = 75.92
e: Main effect of first-order RT on confidence (R+ trials)	Single-trial response times (continuous)	Bayesian mixed-effects linear regression	Mean = −0.15 ± 0.02	Evidence ratio > 4000),
f: Interaction between condition (CR+R+/CR+R−) and confidence on proxy accuracy	Single-trial accuracy (binomial)	Bayesian mixed-effects logistic regression	Mean = −0.11 ± 0.39	Evidence ratio = 1.57
g: Main effect of confidence on proxy accuracy (CR+ trials)	Single-trial confidence ratings (continuous)	Bayesian mixed-effects linear regression	Mean = 0.82 ± 0.32	Evidence ratio = 116.65
h: M-ratio between conditions (CR+R+ vs CR+R−)	Posterior probability distributions (continuous)	Differences between Highest density intervals	HDI = [−1.42, 0.89]	NA

NA, Not applicable.

Under a normative view, confidence is a finer-grained description of the same perceptual evidence that leads to the binary first-order decision and, specifically, correspond to the probability of giving a correct answer given the available perceptual discriminability (Pouget et al., 2016; Sanders et al., 2016). In other words, whereas participants choose between two options in the first-order task, they have the chance to more precisely describe the difficulty of their perceptual experience through confidence reports in the second-order task. In this view, introspection is required to produce accurate confidence reports. But recent results have challenged this standard view of confidence as a description of perceptual evidence by showing that, beyond perceptual evidence, sensorimotor signals associated with the response to the first-order task may also contribute to confidence. At its simplest, this effect is manifest as a negative correlation between first-order reaction times (RTs) and confidence reports (Henmon, 1911; Baranski and Petrusic, 1995), which can be explained by bounded evidence accumulation models (Pleskac and Busemeyer, 2010; Ratcliff and Starns, 2013; Moran et al., 2015). The dependency is strong when accuracy is stressed but is greatly reduced (Vickers and Packer, 1982) or disappears altogether when speed is emphasized, suggesting that the influence of predecisional and postdecisional cues on confidence depends on the task demands (Baranski and Petrusic, 1998). Nevertheless, overall, data from a wide range of recent tasks measuring confidence following discrimination decisions shows that an overwhelming majority of participants present a negative relationship between confidence and decision reaction times (Rahnev et al., 2019). Evidence from comparisons between participants further supports this idea: metacognitive performance was better in participants with large differences in response times between correct and incorrect responses (Faivre et al., 2018).

Beyond behavior alone, Gajdos et al. (2019) showed that confidence increases in the presence of subthreshold motor activity before first-order responses. Plus, we recently showed that α-desynchronization before first-order response (an electrophysiological signature of motor preparation) correlates with confidence over different perceptual tasks (Faivre et al., 2018), that metacognitive performance for decisions that are committed with a keypress is better than that for equivalent decisions that are observed (Pereira et al., 2020), and that sensorimotor conflicts alter confidence (Faivre et al., 2020). Finally, transcranial magnetic stimulation (TMS) directed at the premotor cortex involved in the first-order response was found to affect confidence ratings, suggesting a causal role of action-related signals for confidence (Fleming et al., 2015).

Experimental manipulations that artificially change the process of evidence accumulation have provided strong mechanistic explanations for this relationship (Fetsch et al., 2014; Kiani et al., 2014; Zylberberg et al., 2016). But these manipulations ultimately affected the evidence available to the observer or the process of accumulation itself. Here, we sought to compare confidence judgments and metacognitive performance between conditions that differed only on the sensorimotor information available for the decision, but that were indistinguishable from the point of view of perceptual evidence. We hypothesized that response-related sensorimotor activity carries information useful for confidence judgments, above and beyond the strength of the (perceptual) internal signal. We designed a paradigm in which participants saw visual stimulus that moved alternatively rightward or leftward for 5 s, and rated their confidence in their capacity to discriminate the motion direction that was presented for the longest duration. Following a preregistered plan (https://osf.io/hnvsb/), we compared conditions with and without overt motor discrimination responses and predicted that conditions with overt first-order two-alternative forced choice (2AFC) responses would reveal better metacognitive performance than those without them.

Importantly, we note that the logic of this design relies on the strong assumption that participants committed to a binary decision even in cases with no overt first-order responses, and that the confidence judgments reflected this. We discuss the implications that follow if these assumptions are not met.

## Materials and Methods

*Participants.* Twenty-seven participants took part in this study, of whom 4 had to be excluded (see below). The results we report here correspond to a sample of 23 participants (13 males, 10 females) with a mean ± SD age of 26.7 ± 5 years. All participants had normal or corrected-to-normal vision and no color blindness, and were right handed. Ten participants were tested in Berlin, the rest in Geneva. All received monetary compensation for their time. The procedures were approved by the corresponding local ethics committees, and institutional review board in conformity with the Declaration of Helsinki. Written and signed informed consents were obtained from all participants.

*Procedure.* The experimental task was written in MATLAB (MathWorks) using Psychtoolbox (Brainard, 1997; Pelli, 1997; Kleiner et al., 2007). Stimuli were red (RGBA color, 0.75, 0, 0, 1) or green (RGBA color, 0, 0.75, 0, 1) vertical gratings that drifted sideways. The gratings were formed by a sine-wave function (0.27 cycles/°), drifting sideways at 15°/s, and drawn inside a square (8° height and width), presented at fixation. The green and red stimuli always drifted leftward and rightward, respectively.

**Figure 1.**
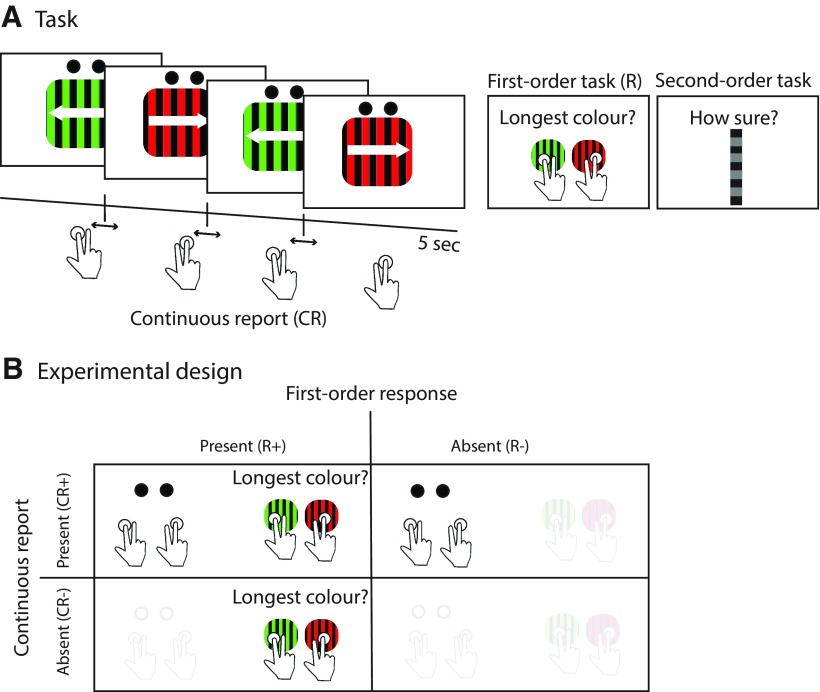
***A***, Task. ***B***, Example trial with both continuous report (CR+) and first-order 2AFC response. On each 5 s trial, two stimuli pairs appeared serially in four consecutive intervals. Participants pressed one of two keys for the entire duration of the trial, tracking the visual presentation (continuous report). Following stimulus offset, participants reported which of the two stimuli had the longest duration overall. ***B***, Experimental design. Each trial was one of the four possible conditions resulting from a combination of first-order 2ACF response and continuous report (CR+R+; CR+R−; CR−R+; or CR−R−). Participants rated their confidence in all conditions. Thus, the task demanded that participants make a first-order 2AFC judgment in every trial, but the corresponding overt action was only present in R+ conditions.

Each 5-s-long trial was divided into four intervals of different durations, during which four red and green stimuli were presented in alternation. The total, summed duration of each pair of same-colored stimulus presentations corresponded to half the trial length (2.5 s) plus or minus a temporal difference determined by a staircase (see below). Further, each single stimulus presentation interval corresponded to half of the sum of the stimulus pair length. The first-order 2AFC task consisted of a duration comparison, followed by a confidence rating (Fig. 1).

To evaluate the effects of overt movement on metacognitive judgments, we asked for two kinds of reports. In continuous-report (CR+) trials, participants pressed two arrow keys using two fingers of their right hand to indicate which of the two colored stimuli was presented on the screen. In this condition, the task was simply to press a key that “tracked” the motion direction of the stimulus. In conditions without continuous report (CR−), participants did not press any keys during stimulus presentation. In trials with first-order 2AFC response (R+) trials, participants did a temporal-summation task. Upon stimulus offset, they indicated with a single key press which of the two motion directions had been presented for a longer period of time (i.e., which of the summed stimulus durations was the longest over the course of the entire 5 s trial). The response keys and hands used for the first-order 2AFC response were the same as for the continuous report. In conditions without the first-order 2AFC response (R−), participants were also required to make a temporal summation decision (the decision was overt in R+ trials but covert in R− trials). Each trial corresponded to one of four possible conditions, combining CR (+, present/−, absent) and R (+, present/−, absent). At the end of each trial, participants rated their confidence in their decision by moving a slider with two keys on a vertical visual analog scale with the ends marked as “very sure” and “very unsure.”

The duration difference was determined separately for CR+ and CR− trials using two independent 1-up, 2-down staircases (updated only following R+ trials). We also ran two pre-experiment staircases of 25 trials each, without confidence ratings, to adjust the difference in duration of the two stimuli for each participant. After the staircases, each participant completed 240 trials in total (60 trials per condition). Trial types were interleaved, and the order of the trials was randomized for each participant. On any given trial, participants were not informed beforehand whether a first-order response would be required. That is, after stimulus offset, participants were either prompted to give a 2AFC on the color corresponding to the longest duration, or were directly prompted to give a confidence rating. Trials were self-paced and the experiment took on average 50 min.

*Design rationale.* As per the preregistration, we hypothesized that response-related sensorimotor activity carries information useful for confidence judgment, above and beyond the strength of the (perceptual) internal signal. We therefore expected that conditions with overt first-order 2AFC responses would be associated with better metacognitive performance than those without motor responses. In the same way, we expected that conditions in which a motor action was paired with the stimulus would also be associated with better metacognitive performance than those without motor responses.

As we anticipated in the Introduction and further elaborate in the Discussion, our analyses and conclusions are only valid if two assumptions are met. First, we assumed that participants committed to a binary decision even in cases with no overt first-order 2AFC responses. Related to that, we also assume that confidence reports in the two conditions are about the same quantity, and that participants reported their confidence in a binary decision that they (covertly) committed to and not, for example, to the uncertainty in the temporal accuracy of their continuous report.

*Termination rule.* Our plan at preregistration was to collect data until we reach a Bayes factor (BF_10_) of either one-third or 3. We started by collecting a sample of 27 participants (four excluded) and examined the data once. With this sample size, we found evidence for the null hypothesis in our main test of interest (the interaction term between confidence and first-order response in the effect on accuracy as modeled by a logistic regression; see Confirmatory analyses below), so we halted data collection.

*Analyses.* We adhered to the exclusion criteria that were preregistered. Four participants were excluded because they did not follow the task instructions (in all cases, they did not press any keys during any of the trials in the continuous report conditions). No further participants were excluded, as none of them had first-order accuracy <60% or >80% in any task; and visual inspection of the staircases revealed no obvious problems. A total of 64 trials (from 17 participants) were excluded because first-order RTs were <200 ms or >5 s.

*Metacognitive performance.* As per the preregistration, we computed metacognitive efficiency (meta-d′/d′) to quantify the capacity to adjust confidence regardless of the first-order task difficulty (Maniscalco and Lau, 2012) using the HMeta-d′ (Fleming, 2017). For that, we scaled confidence judgments for each participant by subtracting from each rating the individual minimum rating and dividing the values by the total range. This procedure effectively “stretched” confidence distributions to fit the interval between 0 and 1 for all participants, thereby eliminating biases between individuals while preserving mean differences between conditions. We then discretized scaled confidence values into four confidence bins. For the MCMC (Markov chain Monte Carlo method) procedure, we used four chains of 10,000 iterations including 1000 for adaptation, no thinning, and default initial values as generated by JAGS (Just Another Gibbs Sampler). Separate hierarchical estimates were computed for each condition. Potential scale reduction factors regarding average M-ratio estimates were equal to 1.02 (CR+R+), 1.02 (CR−R+), 1.06 (CR+R+), and 1.16 (CR+R−). Only the last value for CR+R− indicates a possible lack of convergence, so we refitted the model with 30,000 iterations including 10,000 for warmup, which resulted in scale reduction factors of 1.03 and 1.11, respectively, with no difference in M-ratios between conditions. These values still point to possible converge problems, presumably due to the relatively low number of trials in our sample.

In separate analyses, we estimated the slope parameter in a mixed-effects logistic regression with accuracy as the dependent variable and confidence as the independent variable. Because mixed-effects logistic regression analyses are not affected by subject-wise scaling of confidence (i.e., they include subject-wise random intercepts), we used raw confidence values as independent variables. For all models, we included a by-subject random slope for each of the main effects considered in the model, but not for their interactions. We ran Bayesian sampling of mixed regressions using the *brms* package (Bürkner, 2017, 2018) for all models, we report the estimate and its associated error mean ± error and the 95% credibility interval (CI).

As no first-order 2AFC responses were provided in R− trials, we defined a proxy based on the percept associated with longer key presses during continuous report (i.e., covert first-order response). This allowed us to relate a proxy for first-order 2AFC responses and confidence ratings to compute metacognitive efficiency in CR+ trials.

*Simulations for power estimations.* We aimed at computing the power of our experimental design and analysis strategy. To do that, we estimated the proportion of simulated “experiments” in which we would have found a significant difference between two given conditions with different M-ratios. We used signal detection theory to simulate first-order and second-order responses from 80 trials for each of the 23 participants (see Fig. 4*A*). We set the distributions of the internal signals elicited by the stimuli to be a normal distribution with μ = ±d′/2, σ = 1 (the sign of μ depended on the longer stimulus presented). First-order responses were defined according to an optimal first-order criterion at 0.

Equation 1.1 describes the internal evidence *e*, as follows:

(1.1) $$e=\{\begin{array}{cc} N(\frac{-1}{2},\frac{1}{2}) & \text{if stimulus}=-1\\ N(\frac{1}{2},\frac{1}{2}) & \text{if stimulus = 1} \end{array}.$$

Equation 1.2 describes the perceptual decision *d* given the sampled evidence *e*, as follows:

(1.2) $$d=\{\begin{array}{cc} -1 & \text{if}e<0\\ 1 & \text{if}e\geq 0 \end{array}.$$

Next, to simulate the first-order proxy, we injected randomly distributed noise into the internal signal, sampled from a normal distribution centered at μ = 0 and σ = 0.8.

Equation 2 describes our proxy for internal evidence *e_*proxy given the sampled internal evidence (*ie*), as follows:

(2) e_proxy=e+N(0,0.8).

This led to a correspondence of ∼70% between real and proxy simulated responses, similar to our data. The rationale for adding noise to the internal signal rather than to the binary response variable itself was to preserve the structure of the data: trials with an internal signal closer to the decision boundary are associated with lower confidence and therefore are more likely to cross over the decision boundary as a consequence of adding noise, compared with trials with an internal signal strength that is far from the decision boundary. We obtained the simulated proxy by binarizing the noisy internal signal data based on the position relative to the same optimal first-order criterion placed at 0.

Finally, to simulate confidence ratings, we first added metacognitive noise by adding to the simulated internal signal an amount sampled from a normal distribution centered on 0 and with *m*σ, to achieve an M-ratio ranging from 0.1 to 4.

Equation 3 describes how we obtained degraded internal evidence *e_*degraded

(3) e_degraded=e+N(0,mσ).

Equation 4.1 describes how we assigned the absolute value of *e_*degraded to confidence if M-ratio <1, as follows:

(4.1) confidence=|e_degraded|.

To simulate M-ratio values above 1, we then swapped the identity of the two distributions to make the second-order distributions sharper than the first-order ones. In a separate simulation, we established that these values of added σ corresponded to M-ratio values ranging between 0 and 1.1, which corresponds to the range of M-ratios in our experimental data (see Fig. 3). We set the simulated confidence as the absolute value of the internal signal; that is, the distance to the first-order decision criterion.

Equation 4.2 describes how we assigned the absolute value of *e_*degraded to the internal evidence if M-ratio >1. In this case, confidence was calculated as the absolute value of the internal evidence *e*, as follows:

(4.2) e=e_degraded.

Thus, we added two kinds of noise to the original internal signals, with different meaning. The first type of noise simulated the imperfect relationship between covert/overt responses and their corresponding proxy. The second type of noise simulated the imperfect mapping between the strength of the internal signal at the point of the first-order and second-order decisions, a relationship captured by M-ratio (Maniscalco and Lau, 2012).

We then submitted these simulated data (for 80 trials from 23 participants) to the same mixed-effects logistic regression we used to analyze empirical data. We repeated this procedure 250 times with each combination of M-ratio to estimate the number of times that a significant effect would occur in 250 experiments.

*Data availability.* The code described in the article is freely available online at https://gitlab.com/nfaivre/filevich_metareport/. The code is available as Extended Data. The preregistered analysis plan can be found at https://osf.io/hnvsb/. The raw data for analysis files used to reproduce all figures are available under https://gitlab.com/nfaivre/filevich_metareport/.

10.1523/ENEURO.0326-19.2020.ed1 Extended Data Supplementary Experimental codes, raw data, analysis, and simulation files. Download Extended Data, ZIP file.

## Results

### Descriptive analyses: effects on confidence

**Figure 2.**
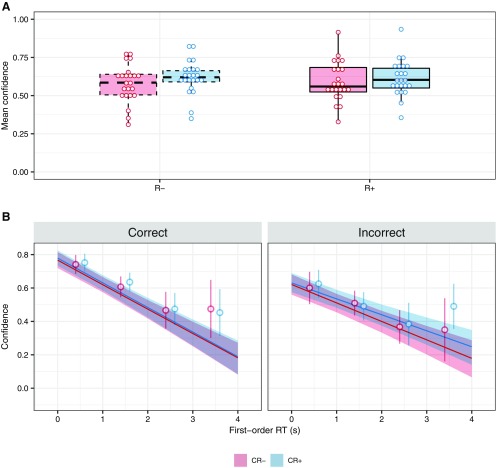
***A***, Differences in confidence judgments between conditions. A 2 × 2 ANOVA on mean confidence judgments revealed that trials with continuous report (CR+) were associated with higher confidence. ***B***, Relationship between first-order reaction times and confidence judgments. Linear mixed-effects regressions revealed that, as expected, confidence judgments had a strong negative relationship with first-order reaction times on a trial-wise level. This relationship was present in all R+ trials (R− trials were not included in this analysis) but was stronger in the subset of correct trials. Regression lines and confidence intervals around them represent the model fit. The model took continuous reaction times as input. For illustrative purposes, we plot open circles and error bars that represent mean ± 95% CI over participants after rounding reaction times and subtracting 0.5 s.

The adaptive staircase procedures successfully fixed performance at ∼71% correct, as follows: mean ± SD accuracy was 72.0 ± 4.2% for continuous report and 72.1 ± 4.6% for no continuous report conditions, with no difference between conditions (*t*_(22)_ = −0.12, *p* = 0.91, *d* = −0.02, BF_10_ = 0.22, Table 1, a). Mean perceptual evidence did not differ across CR+R+ and CR−R+ trials (*t*_(22)_ = 1.75, *p* = 0.09, *d* = 0.36, BF_10_ = 0.54, Table 1, b), indicating that pairing motor information to the perceptual input was not informative for the first-order decision. Next, we tested for mean differences in confidence between all conditions using a linear mixed-effects regression model on confidence. The model included the two experimental manipulations (R and CR) and their interaction as fixed effects, intercepts for subjects as random effects, and a by-subject random slope for each of the factors. We found no interaction between overt first-order 2AFC responses and continuous report (mean = −0.02 ± 0.01, evidence ratio = 0.10, Table 1, c), no strong effect of overt first-order 2AFC responses on mean confidence ratings (mean = 0.01 ± 0.02, evidence ratio = 3.43), but a significant increase of mean confidence in conditions with continuous report (mean = 0.04 ± 0.02, evidence ratio = 75.92, Table 1, d; Fig. 2*A*).

Importantly, to test the hypothesis that the monitoring of first-order 2AFC responses or their underlying processes contributed to confidence, we first established the existence of a relationship between reported confidence and first-order RT. We did so by fitting a mixed-effects linear regression to confidence in trials with overt first-order 2AFC responses (R+), including first-order accuracy, first-order RT, condition (CR+/CR−), and perceptual evidence as fixed effects, random intercepts for subjects, and by-subject random slopes for each fixed effect. As expected, we found a strong main effect of first-order 2AFC RT on confidence (mean = −0.15 ± 0.02, evidence ratio > 4000, Table 1, e), confirming the relationship that has been reported in previous studies (Henmon, 1911; Vickers and Packer, 1982; Baranski and Petrusic, 1995; Patel et al., 2012; Fig. 2*B*). This effect was stronger for correct trials than for incorrect trials (interaction effect estimate: mean = 0.04 ± 0.02, evidence ratio = 46.06). We also found a main effect of accuracy (mean = −0.15 ± −0.03, evidence ratio > 4000) and of perceptual evidence (mean = 0.22 ± 0.06, evidence ratio > 4000), indicating that confidence was higher for correct responses, and in the presence of higher perceptual evidence. However, the model revealed no main effect of condition (mean = 0.01 ± 0.02, evidence ratio = 0.41). No other model parameters were associated with confidence.

Together, these two analyses on mean confidence and the relationship between first-order 2AFC RT and confidence indicate that fast first-order responses were associated with higher confidence, but that response times are unlikely to play a causal role as removing first-order responses altogether had no effect on mean confidence.

### Confirmatory analyses: effects on metacognitive sensitivity

Our first hypothesis was that sensorimotor activity related to first-order 2AFC responses carries information useful for confidence, above and beyond the strength of perceptual evidence. We therefore expected trials without first-order 2AFC responses to be associated with better metacognitive sensitivity (measured as the relationship between confidence and first-order accuracy) than those with responses to the 2AFC task. As we could not calculate response accuracy in trials with no first-order 2AFC responses (R−), we assumed that the percept associated with longer key presses during continuous report corresponded to the covert first-order 2AFC response. Hence, we limited this analysis to CR+ trials alone. In CR+R+ trials, this proxy based on continuous report predicted the actual first-order 2AFC response in 65.5 ± 8% of trials (ranging between 50% and 79.6%). For CR+R+ trials, we confirmed that response predictability based on the stimulus (i.e., the longest stimulus presented) and proxy (i.e., key pressed the longest) was significantly higher than that based on the stimulus alone (difference in Bayesian information criterion = 2.9, χ^2^ = 10.04, *p* = 0.002). In other words, the proxy, derived from the motor-tracking behavior, consistently added predictive power to the stimulus presented, which was already above chance (∼71%) for the covert response provided in the first-order 2AFC task. This is why, despite low predictability scores, we proceeded with this analysis as per our preregistered plan and pursued alternative ways to analyze the data in the Exploratory analyses section described below.

We then compared metacognitive sensitivity between conditions with and without first-order 2AFC responses. It is only possible to estimate metacognitive sensitivity in R− trials if they are also CR+. In other words, we required the continuous report from CR+ conditions to estimate metacognitive sensitivity in cases of no first-order 2AFC response (R−). Therefore, we built a mixed-effects logistic regression for proxy accuracy that included condition (CR+R+/CR+R−) and confidence and their interaction as fixed effects, as well as subject-wise random intercepts, and random slopes for both confidence and condition. If metacognitive monitoring is affected by the presence of first-order 2AFC responses, this should manifest as a significant interaction effect between confidence and the presence of a first-order response: the relationship (slope) between confidence and proxy accuracy should be stronger for trials with first-order responses than for those without them. Against our expectations, but in line with the results on mean confidence reported above, we found no interaction effect (mean = −0.11 ± 0.39, evidence ratio = 1.57, Table 1, f). On the other hand, a main positive effect of confidence (mean = 0.82 ± 0.32, evidence ratio = 116.65, Table 1, g) indicated that the likelihood that the proxy was the correct answer increased with confidence and thus, simply put, that participants had some metacognitive access to their response accuracy.

The estimation of M-ratio (meta-d′/d′) using the HMeta-d′ toolbox (Fleming, 2017) revealed consistent results, as we found no differences between conditions in the M-ratio estimates (R+: M-ratio = 0.22, HDI = [0.12, 0.42], R−: M-ratio = 0.25, HDI = [0.10, 0.45]; difference between conditions: highest-density interval (HDI) = [−1.42, 0.89], Table 1, h).

Our second preregistered hypothesis was that metacognitive performance between conditions with and without continuous report would differ because the key presses in the continuous report constitute an additional source of information for confidence responses. To test this hypothesis, we followed two approaches. First, using the same approach as above, we measured metacognitive sensitivity as the relationship between confidence and first-order 2AFC accuracy. Here again, a main effect of confidence on accuracy (mean = 2.51 ± 0.37, evidence ratio > 4000) suggested that participants could monitor their performance. However, we found no interaction between confidence and condition (mean = 0.13 ± 0.35, evidence ratio = 1.77), indicating that this effect was comparable with and without continuous report. This analysis included only trials with overt first-order 2AFC responses (R+), so it was possible to measure metacognitive accuracy with standard methods. Thus, we also estimated M-ratio (meta-d′/d′) in trials with and without continuous report. Again, and consistent with our regression analyses, we found no differences between conditions in the M-ratio estimates (CR+: M-ratio = 1.06, HDI = [0.83, 1.32], CR−: M-ratio = 0.98, HDI = [1.24, 0.77]; difference between conditions: HDI = [−0.27, 0.40]).

Thus, our data revealed no differences in the relationship between confidence and first-order 2AFC accuracy between conditions.

To measure the effect of first-order responses (CR+R+ vs CR+R−), we relied on a proxy as the best informed guess for the covert first-order response; but the proxy was noisy and corresponded to the overt first-order response only for ∼65% trials over all participants. In other words, with this analysis we injected noise into our first-order response, which might have in turn affected both the value of the confidence × condition interaction estimates and our ability to find robust effects. To examine whether this was the case, and to what extent this affected our results, we simulated data from 250 “experiments” to compare the power of the logistic regression analysis based on the simulated first-order response and on the degraded first-order 2AFC proxy.

The results of these simulations (Fig. 4*B–D*) validated our analysis strategy. First, we found that power between the two analyses did not differ for values far from the diagonal (i.e., pairs of M-ratios with large differences between them). Second, and crucially, we found that even in regions where the proxy analysis fared worse (i.e., had lower power), power reductions in the range of 0.1–0.3, may partially, but not completely, explain our null results. This reduction in power reveals that we cannot strictly rule out that null effects in the proxy-based analyses are not due to low sensitivity. The loss of power is, however, intrinsic to a paradigm like ours, where the identity of covert responses is indirectly inferred and could only be avoided in principle if we had a lossless proxy with perfect accuracy. Interestingly, on the other hand, power estimations for the proxy-based analyses showed a somewhat smoother pattern than those from actual responses. This result, presumably an effect of having an additional source of Gaussian noise, may be an unexpected advantage of the proxy-based analysis in preventing false inferences.

### Effects of experimental manipulation

It has been recently shown that the variability in the stimuli presented may lead to inflated estimates of metacognitive performance (Rahnev and Fleming, 2019). To assess whether this was a problem in our data, we ran two separate analyses. We compared the range and SD of the stimuli presented to each participant in CR+ and CR− conditions (we did not compare R+ and R− conditions because these were yoked to their corresponding condition based on CR). In both cases, we found strong evidence for the null hypothesis (range of stimulus strengths presented: χ^2^ = 0.1075, *p* = 0.743, difference in Bayesian information criterion = −8.56, BF_10_ = 0.014; SD of stimulus strengths presented: χ^2^ = 0.01, *p* = 0.752, difference in Bayesian information criterion = −10.86, BF_10_ = 0.004), indicating that our estimates of metacognitive sensitivity are not inflated due to stimulus presentation.

### Exploratory analysis: machine learning tools to predict first-order responses

We considered that the relatively low predictability of the continuous report-based proxy could be poor due to its simplicity: the proxy was based on nothing more than the longest reported percept in each CR+ trial. To extract as much information as possible from CR+ trials, we leveraged standard machine learning (ML) algorithms to predict first-order responses from CR+ information. First, for each CR+R+ trial we extracted features including the number of transitions in the key press response, the identity of the first and last stimuli shown and keys pressed, the total time with correct and incorrect key presses, and the delay between each stimulus presentation and the response. Using the scikit-learn module in Python (Pedregosa et al., 2011), we then trained three different classifiers on the data pooled over all participants using the following leave-one-out cross-validations: logistic regression, naive Bayes, and k-nearest neighbors. Their accuracy, based on the confusion matrix on CR+R+ trials revealed low overall predictability: 0.63, 0.61, and 0.64, respectively. These relatively low values are comparable to those of our simple proxy, and we therefore did not carry out any further analyses with the ML-based predictions.

## Discussion

The past years have seen a growing interest in elucidating the sources of information that contribute to confidence judgments, as a window into potential computational processes that allow us to monitor our own thoughts. Converging evidence from very different experimental paradigms suggested that confidence is modulated by motor information concurrent with the first-order response (Vickers and Packer, 1982; Vickers et al., 1985; Baranski and Petrusic, 1995, 1998; Ratcliff and Starns, 2013; Fetsch et al., 2014; Kiani et al., 2014; Moran et al., 2015; Zylberberg et al., 2016; for review, see Anzulewicz et al., 2019). Here, we set out to directly investigate this possibility. Concretely, we used a temporal-summation metacognitive task and asked whether committing a motor response associated with the response affected corresponding confidence judgments. Participants were instructed to rate their confidence in the accuracy of a binary decision both in trials with covert and overt first-order responses.

### Effect of first-order responses on confidence ratings

As a precondition for our analyses, we first replicated what several studies had shown before (Fleming et al., 2010; Patel et al., 2012): in trials with overt first-order 2AFC responses (R+), reaction times to the first-order task showed a clear negative correlation with reported confidence. Based on these results alone, our data are in principle compatible with the hypothesis that first-order responses influence reported confidence. Crucially, we tested this hypothesis directly by comparing two conditions of the task that differed in whether participants had overtly responded with a key press to the first-order 2AFC task (CR+R+), or if their response remained covert (CR+R−). We first compared conditions in terms of average confidence judgments. Against our expectations, and despite the strong correlation between first-order reaction times and confidence, we found that absolute confidence judgments did not vary with the presence or absence of overt responses.

**Figure 3.**
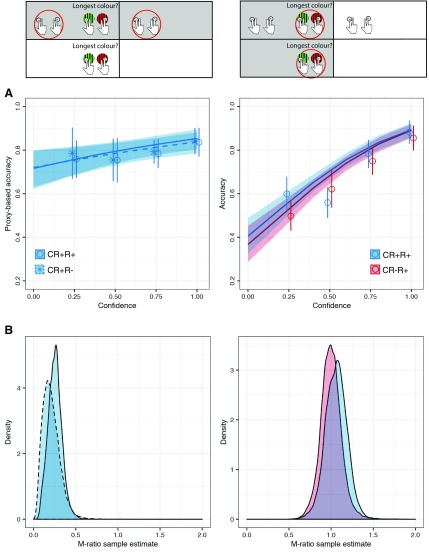
Differences in metacognitive performance between conditions. ***A***, Metacognitive sensitivity quantified with a regression model on accuracy versus confidence. Estimated regression curves from the proxy for first-order 2AFC response (left) and overt first-order 2AFC response (right). The presence of a first-order 2AFC response did not affect the relationship between confidence and the first-order accuracy of the proxy. Open circles and error bars represent the mean ± 95% CI over participants after rounding confidence ratings. ***B***, Metacognitive efficiency quantified with M-ratio. As in ***A***, we found no evidence that either giving an overt first-order response (left) or pairing an action to perceptual input (right) improved metacognitive efficiency. The insets above the panels highlight (in gray) which trials were used for each of the analyses.

To further investigate the effects of overt responses, we then examined an important aspect of confidence judgments, namely their precision. That is, we considered that while participants may not have felt in general less confident in trials with covert responses, the quality of confidence judgments might have been degraded, resulting in a decrease in metacognitive performance relative to trials with overt responses. Measures of metacognition (metacognitive sensitivity, based on logistic regression and efficiency, based on M-ratio) rely on relating trial-wise confidence to accuracy. As the identity of covert responses remained latent by design, we inferred them relying on a proxy based on continuous reports (CR+). Concretely, we considered the percept with the longest key press as a proxy for both overt and covert first-order 2AFC responses. We then compared metacognitive sensitivity and efficiency based on the relationship between confidence and the proxy for responses. Here, mirroring the results from the analysis of absolute confidence values, we found no effect of overt first-order 2AFC responses. A concern with this analysis is that the proxy only corresponded to actual (overt) responses in an average of ∼65% of trials, which resulted in a systematic underestimation of metacognitive performance (Fig. 3, compare dashed blue lines between panels). However, as the proxy predictive power does not differ across conditions, comparisons of metacognitive performance across conditions are still legitimate.

**Figure 4.**
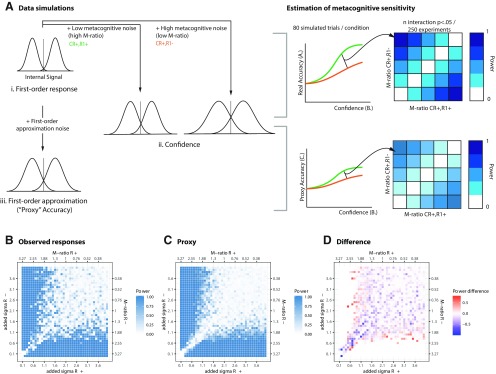
Power simulations. ***A***, Data simulation strategy. We considered two conditions (in this case, CR+R+ and CR+R−) expected to differ in M-ratio. For each 1 of 250 experiments, we simulated 80 trials per condition, drawing three values for each of the “real” internal signal (top left row, i), a noisy confidence estimate (internal signal + metacognitive noise for each of two conditions; middle row, ii) and a value for the noisy proxy (bottom left row, iii). We fed the simulated trials into a logistic regression model, and determined the power of our analysis [i.e., the proportion of “experiments” in which the interaction term (representing a difference in metacognitive sensitivity between conditions) was significant (right)]. ***B***, ***C***, Results: power estimations for the analysis based on actual responses (***B***) and for proxy-based responses (***C***). ***D***, The power difference between ***B*** and ***C***. There are no differences in power when differences in M-ratios between two conditions are large (regions away from the diagonal), whereas there are small decreases in power for the proxy-based analysis for combinations of M-ratios that are closer to the diagonal.

We note that in these analyses we assumed that participants had followed our instructions to rate confidence in the binary decision for both committed and omitted responses. We discuss in the Limitations section the implications for our conclusions if participants did not follow these instructions.

### Effect of continuous report on confidence ratings

In a factorial design, we also tested for the effect of continuous report paired to stimulus presentation on confidence judgments. Over conditions with and without first-order 2AFC responses (both R+ and R−), we found a consistent increase in confidence following continuous report (CR+ vs CR−) despite no changes in first-order performance. Previous studies have shown that different factors can affect first-order and second-order performance independently. These factors include experimental manipulations like changes in stimulus variability (Spence et al., 2016) or sensory reliability (Bang and Fleming, 2018), pharmacological silencing of different brain regions (Stolyarova et al., 2019), as well as the existence of subthreshold motor activity (Gajdos et al., 2019), differences in movement parameters (Faivre et al., 2020), or voluntary control (Charles et al., 2020). Our study adds a novel kind of manipulation, namely, the occurrence of motor responses, to the list of experimental manipulations that affect confidence but not first-order accuracy. Alternatively, higher confidence ratings may result from criterion shifts. In fact, our model comparison showed that motor behavior could explain first-order 2AFC responses over and above perceptual evidence, suggesting that key presses in continuous report conditions were an additional source of information available for both the perceptual task (first-order) and the confidence task (second-order). With additional sources of information, participants may place their second-order criteria more liberally, resulting in higher confidence ratings.

### Differences with the existing literature

To the best of our knowledge, this study is unique in that the effect of motor components on confidence was investigated by completely removing the first-order 2AFC response in some conditions, and replacing it instead with actions paired to the stimuli presentation. As a consequence, we never required participants to provide explicit responses in covert response conditions. Instead, we inferred them through participants’ continuous report. Other studies have addressed the same question by using different experimental manipulations, which can be broadly grouped as following one of three approaches. A first set of studies has asked participants to rate the confidence of observed, rather than committed actions, by letting participants observe only first-order RTs in some of the experimental conditions (Patel et al., 2012; Vuillaume et al., 2019) or both RTs and stimuli (Pereira et al., 2020) before making the confidence judgment. A second group of studies has manipulated the perceptual evidence accumulation process (Fetsch et al., 2014; Kiani et al., 2014; Zylberberg et al., 2016). A third group of studies have instead manipulated the timing of the confidence judgment relative to that of the first-order response (Siedlecka et al., 2016; Wokke et al., 2020). Finally, a fourth approach consists in directly manipulating motor signaling either physiologically using TMS (Fleming et al., 2015) or behaviorally by instructions (Faivre et al., 2018; Palser et al., 2018). Here, we followed the novel strategy of removing first-order responses and instead inferring them from stimulus-coupled responses. Against what has been reported in the literature and our expectations, we found that bypassing first-order 2AFC responses had no observable effect on metacognitive performance.

Our results also revealed that continuous motor responses contingent to perceptual evidence significantly increased confidence. A brief review of the literature reveals that motor activity impacts confidence biases and metacognitive performance distinctively, with large variations across experimental paradigms. On the one hand, our results are in line with what was reported by Gajdos et al. (2019), who found that subthreshold motor activity before a decision increased confidence bias, with no impact of metacognitive performance. Other experimental manipulations produced the converse effect, namely, a modulation of metacognitive performance with no change in confidence bias. This includes the comparison of confidence in committed versus observed decisions (Pereira et al., 2020), and confidence under high or low sensorimotor conflicts (Faivre et al., 2018). Using a similar design comparing prospective and retrospective confidence judgments, Siedlecka et al. (2016) found that both confidence bias and metacognitive performance increased in the presence of action-related signals. This set of mixed results questions the functional relevance of motor signals and suggests that the relationship might be more complex than previously thought. We speculate that the computation of confidence may be flexible and may largely depend on the information that is globally available. In all previous studies, to the best of our knowledge, participants had access to some form of first-order reaction time information, at some point in time during the trial, as follows: either through observation from the third-person perspective, directly after the confidence report, or through simple access to reaction times produced under experimentally manipulated motor signals. In some conditions of our experiment, instead, responses were completely absent and may have shifted participants’ global strategies for the computation of confidence. In other words, we contest that while first-order reaction time information is, under some experimental settings, used by participants to generate a confidence judgment, when motor information is not available at all, it may be replaced by other, equally precise sources of information, closer to the strength of evidence [e.g., the probability of being correct (Sanders et al., 2016), the internal signal noise (Navajas et al., 2017), and the evidence in favor of the chosen response alternative (Peters et al., 2017)]. This admittedly speculative account is in line with a previous study (Reyes and Sackur, 2014) showing that an introspective report in a visual search task (i.e., subjective reports about the number of items scanned, or the time required to scan them) may rely on different sources of information depending on the task context. This kind of introspective flexibility may explain our capacity to form confidence estimates about decisions that are not directly linked to a transient motor action, for instance, when controlling a brain machine interface (Schurger et al., 2017) or when making global confidence judgments in ecological contexts (Rouault et al., 2019).

### Limitations and future directions

A limitation of our design lies in the capacity to identify covert first-order 2AFC responses from continuous reports. Voluntary key presses paired to the stimuli shown on the screen were a relatively poor predictor of first-order responses, and our simulations revealed that this led to lower statistical power in the proxy-based analyses, compared with analyses based on overt responses. However, we argue that the approach is promising given that future lines of research might take this first step further to develop “no-report” paradigms where covert decisions can be unequivocally inferred without a margin for error. Potential approaches include either eliciting an automatic response like the optokinetic nystagmus (Frässle et al., 2014), instead of a voluntary one like the key presses we used here; requiring voluntary key presses in highly trained participants, leading to low latencies between perception and response; or inferring responses through covert attention measured using steady-state visual evoked potentials (de Heering et al., 2019). Another limitation is the use of adaptive staircase procedures throughout the experiment. While maintaining task difficulty constant across trials, conditions, and participants is important to finely estimate metacognitive performance (Rahnev and Fleming, 2019), it also may hinder the relevance of sensorimotor signals as informative cues regarding the difficulty with which a decision was made (Kiani et al., 2014). Thus, a possibility is that sensorimotor signals are more potent cues for confidence estimates under fluctuating task difficulty.

Finally, and importantly, we note that our interpretation of the results relies on the following two assumptions about trials without overt first-order 2AFC responses: first, we assume that participants committed to a binary decision on CR+R− trials, although they were not asked to overtly provide one; and, second, we assume that participants reported their confidence about the binary decision on both CR+R− and CR+R− trials. In other words, we assume that the only differences between CR+ and CR− conditions, and between R+ and R− conditions were the manipulations that we induced experimentally (continuous responses and first-order 2AFC responses, respectively), but that these differences had no impact on the cognitive processes that took place to produce the confidence judgments. If these assumptions were not met, our interpretation would not be valid.

If participants did not commit to a covert decision, despite our instructions and experimental design, this could imply that confidence ratings reflect different quantities in CR+R+ and CR+R− trials, making the comparison between conditions problematic. Specifically, while confidence in CR+R+ trials presumably reflects the probability that a binary decision was correct, given the external evidence (Pouget et al., 2016; Sanders et al., 2016; Fleming and Daw, 2017), it might reflect other quantities like the precision of the internal representation (Meyniel et al., 2015; Meyniel and Dehaene, 2017). Further, previous studies have shown that committing to a binary decision can affect the internal representation of the evidence, both at the first-order level (Stocker and Simoncelli, 2007; Luu and Stocker, 2018) and second-order level (Navajas et al., 2016, Peters et al., 2017). Because the internal representation is modified by a decision, confidence in two conditions that differ in whether a decision has been made may plausibly also differ in the biases and additional evidence accumulation taking place, making a direct comparison of confidence between R+ and R− trials potentially problematic.

Nevertheless, we have reasons to believe that our assumptions are, in fact, justified. First, by instruction, we asked participants to make a decision (and rate their confidence in its accuracy) even in cases where they were not prompted to explicitly provide the answer. Additionally, participants did not know beforehand whether, on each trial, they would have to provide a first-order response. Then, from the participants’ point of view, R+ and R− trial types were indistinguishable until the point of stimulus offset.

Notwithstanding these limitations, we note that we found a clear null effect on absolute confidence differences between conditions with covert and overt first-order 2AFC responses. This result, which is not contaminated by imprecision in our identification of covert first-order 2AFC responses, more strongly argues for our interpretation that motor signals need not be used in metacognitive monitoring.

### Conclusion

Identifying the sources of information that feed into confidence judgments is a core issue in metacognition research. This study suggests that, while confidence judgments correlate with first-order reaction times, this relationship may be merely correlational, as removing the execution of first-order decisions altogether had no visible impact on confidence or metacognitive performance. By contrast, motor actions paired to stimulus presentation boosted confidence, but not metacognitive performance. These results, then, do not support the emerging idea that metacognition relies on the monitoring of sensorimotor signals and call for further research to find the underpinnings of metacognitive judgments.

## Synthesis

Reviewing Editor: Bradley Postle, University of Wisconsin Decisions are customarily a result of the Reviewing Editor and the peer reviewers coming together and discussing their recommendations until a consensus is reached. When revisions are invited, a fact-based synthesis statement explaining their decision and outlining what is needed to prepare a revision will be listed below. The following reviewer(s) agreed to reveal their identity: Jérôme Sackur.

Reviewer #1

1 Summary

═════════

The paper asks the simple question “do overt actions linked to the stimulus improve metacognitive efficiency?” and claims to provide a clear negative answer based on an innovative experimental paradigm where participants perform a confidence judgment on a perceptual decision task, after having tracked the stimulus (CR+) or not (CR-) and emitted a decision (R+) or not (R-).

The paper is as a whole well written and clear. The paradigm is indeed innovative, and the analyses, more generally the strategy, is sound with respect to modern standards (the analytical tools are state of the art, the study is preregistered). Yet I have a few reservations concerning the argumentation, and I think that some improvements in this respect could be helpful:

1/ The literature reviewed is confined to very recent papers, and while it is obviously good to be up-to-date, one should not miss important older papers. With respect to the link between RTs and confidence judgments, and the locus of confidence computations more generally, I think there are useful ideas and results in Baranski and Petrusic pioneering studies, in Moran et al., 2015, in Pleskac & Busemeyer, 2010 and in Ratcliff & Starns, 2013 for instance, or even, let’s play the archaeologist, Vickers & Packer, 1982 Vickers et al., 1985 - quite relevant on the link between RTs and confidence.

2/ It seems to me that the discussion of the merits and limits of the paradigm is a bit short. The paradigm is presented as a factorial 2 (continuous tracking of the stimulus (CR): present / absent) X 2 (Overt response (R)): present / absent) design. But in fact, it cannot be analyzed as a factorial design except for one rather peripheral analysis (confidence bias analysis), and the critical analyses are simple comparisons not based on the same response.

The real problem lies in the fact that the authors claim that the real import of their proof comes from the part where they use the tracking behavior as a proxy for an unobserved decision. Below I detail why this interpretation of their paradigm is questionable, even beyond the fact that tracking accuracy and decision accuracy do not correlate very well. I think that the critical problem lies in the fact that the very notion of a decision (and the theory of metacognition that goes with it) is tied to the idea that in deciding, participants commit themselves to one categorical option as opposed to another. This is why models of decisions have thresholds or bounds. Tracking is a form of continuous response that does not entail such commitment. In the data that the authors present, I find no evidence of a decision in this categorical sense when there is no overt decision. Of course tracking and decision accuracy correlate (because perceptual sensitivity is involved in both), but this is absolutely no proof of the reality of a decision in the absence of a decisional response. To me, the argument: 1/ remove the empirical evidence for the decision and hope that participants still commit to a decision; 2/ use an objectively non-decisional behavior as a proxy for the hypothesized decision; 3/ apply standard metacognitive reasoning on it, is fragile at best. (As an example of a Bayesian analysis of the subtle link between categorical decisions and continuous responses, see Stocker and Simoncelli, _A Bayesian model of conditioned perception_, 2008.)

Some inconsistencies and ambiguities in the vocabulary derive from the above conflations.

In all, I think that the paper is very interesting and very well executed as far as the technical parts go, but that it could be strengthened with respect to the precision of the argument.

2 Major remark

══════════════

I have only one major reservation, that I already more or less explained above, and that I will try to expound on now to be clearer, and specify what I think might be another interpretation of the results. So as to test whether metacognitive accuracy is impacted by the presence of an overt decision, the authors use as a proxy of decision accuracy the tracking behavior (notably ll 313 sqq). But their “proxy” is a valid measure in itself: it is a measure of tracking accuracy (albeit binarized, which may not be necessary). They could have had a confidence judgment on this tracking task. It would have made sense to test whether metacognition for this task is impacted by the presence of a decision - à la Stocker & Simoncelli, 2008, but at the metacognitive level. But now, they compute metacognitive efficiency on a tracking task, based on a confidence judgment that is relative to the decision. So there is some crossover between the tasks at the first and second order. If you give instructions to participants, you should expect that they somehow try to follow them. When you ask them to track the stimulus, you don’t ask them to make a categorical decision; and when you ask them to report the confidence they have in their decision, you don’t ask them how confident they are on their tracking.

It is standard to use proxies, but in doing so, one must realize the alternative and the implications. In fact, due to the above crossover, the situation is completely symmetrical. The decision to take tracking as a proxy for decision is formally on a par with the decision to take decision confidence as a proxy for tracking confidence.

Thus, we could formulate the results the other way around: why don’t call the confidence measure a proxy for the confidence judgment on the tracking task? In that case, the conclusion would be: metacognition efficiency for duration tracking is not impacted by the presence of an overt decision. A conclusion clearly different from “metacognition for decision is not impacted by the presence of first order behavior”. Note that crucially, this other formulation nowhere uses the traditional in the last decade or so, decision related, concept of metacognitive efficiency that is the target of this paper.

Furthermore, I would argue that using confidence in decisions as a proxy for confidence in tracking is more justified than to use tracking as a proxy for decision. Why? Because at the first order level we have incontrovertible behavioral evidence that tracking and decision are opposed on the crucial commitment parameter. We don’t have such strong evidence at the second order level. Ignoramus arguments are weak and one could retort that it may be even worse at the second order level..

Compared to the above analysis, the “second preregistered analysis” (344: CR+R+ / CR-R+) seems completely sound to me.

Such consideration would lead to a reinterpretation of the results: The conclusion drawn from the CR+R+ / CR-R+ comparison would be: “the presence of an overt tracking behavior on the very stimulus on which a decision is based does not impact metacognitive accuracy” and the conclusion drawn from the CR+/R+ / CR+R- comparison would be: “the presence of an overt decision on the very stimulus which is the basis of some tracking behavior does not impact metacognitive accuracy for this tracking”.

With this reinterpretation, the paradigm of the paper would squarely fit in the “third class” of studies reviewed (l. 502), and it would be very far from a “no-report” paradigm.

So I think that the discussion about tracking behavior being a mediocre but still usable proxy for decision misses the point. It is not a matter of quantitative adequacy, but of theoretical distinctiveness. Response times and precision are usually correlated (except when there is a speed-accuracy trade-off), but they are different construct. A DDM clearly explains why we have a correlation, and it would show to what extent one could use one as a proxy for the other.

3 Minor remarks

═══════════════

3.1 Abstract

────────────

The fact that “across a broad range of tasks, trials with quick reaction times to the first-order task are often judged with relatively higher confidence than those with slow responses” is of course no proof that “confidence could be informed by a readout of reaction times in addition to decision-monitoring processes", but it is not even really suggestive of that, as it is quite obvious that most models of decision will have parameters that impact both response times and confidence, without the later having to be in any way based on the former (see Vickers, 1985; Pleskac & Busemeyer, 2010; Ratcliff & Starns, 2013).

I find the last sentence of the abstract a bit unclear. I don’t understand why the rejection of the null hypothesis (as the preview of the results has it) would have rejected the notion that the relationship between first order and confidence judgments is “correlational”.

3.2 Significance statement

──────────────────────────

I would remove the reference to the brain. In metacognition, it’s the mind that monitors itself. The brain might be said to monitor itself when someone is looking at his / her own real time fMRI or EEG.

The assertion that only post-decisional signals is “truly metacognitive” is a bit rash. I think it conflates retrospection with metacognition. A judgment might be authentically metacognitive (second order) while being made as the decision unfolds. Cf Fleming & Daw, 2017.

3.3 Introduction

────────────────

l. 44 reference to “perceptual judgment” is too narrow, as it also applies to other tasks, as the authors state themselves on ll. 52 sq.

l. 77 The sentence “A drift-diffusion model explained this effect by showing that the accumulation of perceptual evidence is constrained by first-order decisions.” is unclear. First, for a reader not acquainted with Pereira et al., 2018, it is unclear how one could provide a DDM for an observed decision. Is it a DDM of the confidence judgment RT? Otherwise I don’t really understand how “perceptual evidence accumulation” could not be related to first order decision? In the sequential sampling framework isn’t perceptual evidence accumulation the crucial process of first order decisions?

3.4 Material and methods

────────────────────────

The psychophysical description, while probably not very important, is incomplete. For instance, what is a “maximum luminance” “sinusoidal grating”? What is the speed of the drift?

It might be useful at some early point to specify that the task is a duration comparison.

Similarly, it would be very useful to learn whether the design was randomized or blocked very early. In fact, it is nowhere stated, but we need to know either how participants are cued for each conditions if it is a randomized design and block duration and counterbalancing if it is a blocked design.

l. 149 I have a real methodological worry concerning R- trials (trials with “covert decision”). The authors state: “In conditions without first-order response (R-), participants were also required to make a temporal summation decision (the decision was overt in R+ trials but covert in R- trials).” But how can we be sure that participants do as they are asked? For all we know participants might not do anything in this case and use the confidence scale to report their confidence in their tracking performance; or use the confidence scale as a means for reporting their first order response... With so little knowledge about whether participants are or are not committing to a decision in R- blocks, even a a qualitative debriefing would be useful.

As for the use of Fleming’s hierarchical Bayesian toolbox for the estimation of meta-d’/d’, it would be useful to have some details concerning the estimation procedure: number of chains, of samples, length of adaptation, starting values (all that for the MCMC procedure). Then we would need some information about the quality of the estimation (at least Gelman-Rubin tests). Most importantly we need to know the hierarchical structure when comparing conditions (eg, l 361, do the authors do a separate hierarchical estimate for each condition, or do they use a regression, with only one estimate?) Most of that could be in SMs.

3.5 Analyses

────────────

I don’t quite understand the interim conclusion l. 281: “Together, these results indicate that fast first-order responses were associated with higher confidence, but that response times are unlikely to play a causal role as removing first-order responses altogether had no effect on confidence.” As I understand it the last analysis before this sentence includes RTs, so how could it prove anything about the causal role of RTs? I think that the authors oscillate between treating the continuous response as a first order response (they need that when there is no other first order behavior) or an accompaniment of the first order decision (they need that so that they can test its causal impact). This is fine, but here they seem to imply that while their model always include RTs, they can consider condition (CR+/CR-) as the difference between RT present and absent...

l. 299 Perhaps some ambiguities could be resolved by using the phrase “first order behavior” for the continuous tracking response. When I read the sentence: “We therefore expected conditions with overt first-order responses to be associated with better metacognitive sensitivity (measured as the relationship between confidence and first-order accuracy) than those without motor responses” my first impulse is to find that incoherent as there cannot be any first order accuracy when there is no first order response. And the problem is sometimes even worse when the first order tracking behavior is used as a proxy for the absent first order response.

ll 301sqq The factorial design seems neat, but still, the authors cannot eat their cake and have it: in case when there is no first order decision and when they use the tracking behavior as a proxy for the decision, they cannot still compare the effect of tracking present vs absent on metacognition efficiency. Unless they use some magical trick that I don’t understand. In other words, What is the use of the tracking absent / first order decision absent in the estimation of metacognitive efficiency?

The transition on line 363 is a bit surprising, because it comes just after the CR+R+ / CR-R+ analysis that is not based on any proxy. The wording of the transition to this part could be better formulated. More generally, I find the presentation of these simulations not optimal. It was already presented in abstracto at the beginning of the Analysis section, but it is only here that one can really understand their justification. And it does not seem crucial to the argument. I would put the whole of it in an appendix / supplementary materials section.

Similarly, the very short ML paragraph would comfortably sit in a supplementary material section.

3.6 Discussion

──────────────

I am not really convinced by the explanation of the increased in absolute mean confidence in the CR+ condition compared to the CR-, based on “attentional demands”: why increasing attention should increase confidence? Do we have some previous literature on that? If anything, Rahnev et al., 2013 find the opposite. In a sense, I might expect that all things equal, increased attention might increase metacognitive efficiency. But why should it make participants overconfident?

l. 524 Reyes & Sackur, 2014 revolves around the notion that introspection is flexible and tries to makes the best of whatever information is available (perceptual, decisional, motor).

l. 546 shouldn’t it be “overt” instead of “covert”?

4 Very minor remarks

════════════════════

Legend of Figure 2. What is the difference between “mean confidence” and “confidence” on the y-axis of panel A and B. I think that “raw RT” would be clearer than “continuous RT” (all the more than there is a continuous duration task in some conditions...)

In Vuillaume et al., 2019 in some conditions participants observe the behaviors and have access to the stimulus, contrary to what is implied on line 500.

I always sign my reviews

Jérôme Sackur

Reviewer #2

The authors hypothesize that a readout of reaction time could lead to confidence judgments, in addition to monitoring of internal decisional processes. I’m a big proponent of trying to uncover what exactly goes into confidence judgments, so I am glad to see this type of work being done. However, my enthusiasm is somewhat dampened here due to some issues which I describe below.

Primarily, I am a little concerned about the implied novelty of this manuscript. Only one citation of Kiani et al., 2014 exists, but it does not appear to inform the formation of the present hypotheses. This is interesting because Kiani et al.’s paper is literally titled “Choice certainty is informed by both evidence and decision time” – seemingly a direct precursor to the present manuscript’s goals. Additionally, no citations exist of Fetsch et al., 2014 or Zylberberg et al., 2016, both of which use this “accumulation time” model to successfully explain seemingly counterintuitive findings in confidence judgments. In fact, the latter (Zylberberg et al.) explicitly investigates how stimulus volatility and reaction time together lead to the construction of confidence.

In addition to the inadequate literature review, I have a few theoretical concerns about the project.

1. The staircase procedures used throughout the block mean that metacognitive sensitivity will be over-estimated (Rahnev & Fleming, 2019). While the authors acknowledge this possibility, they do not investigate how staircase variability changed from one block to the next or how such variability could have impacted metacognitive sensitivity estimates.

2. Relatedly, this is especially important given that confidence judgments have been shown to depend on absolute as well as relative evidence favoring a decision (e.g. Zylberberg et al., 2012; Maniscalco et al., 2016; Samaha et al., 2016; Peters et al., 2017; Odegaard et al., 2018). “Absolute evidence” has been defined differentially as contrast or % of coherently moving dots, among other variants. This means that the continuous staircase changes are not only affecting meta-d’/d’, but also potentially confidence judgment magnitudes themselves in ways unaccounted for by the analyses or signal detection theoretic power simulations. It is unclear how the authors may deal with this, as “perceptual evidence” e.g. line 278) appears to refer to relative and not absolute evidence.

3. I am concerned about the assumption that “the percept associated with longer key-presses during continuous report corresponded to the covert first-order response” (line 303-304). In the simulation, the authors state that the correspondence between overt and covert responses was about 70%. This suggests that at low evidence levels, the effect of noise is considerable. This is confirmed by the fact that “response predictability based on the stimulus ... and proxy ... was significantly higher than based on the stimulus alone” (lines 307-309). I worry that this lack of correspondence may unduly inflate meta-d’/d’ judgments, because the participant’s d’ as defined via this proxy measure may be higher than the d’ actually experienced by the subject. That the staircase showed matched performance between R+ and R- trials does not fix this: if proxy-defined d’ in R- = reported d’ in R+, but *true* d’ is actually lower in R-, then d’ in R- will seem artificially inflated, thus reducing the meta-d’/d’ ratio because the too-high d’ is in the denominator. The authors could test this by comparing proxy-defined d’ in R+ and R- conditions – Figure 3a seems to suggest there may be a small but significant effect. If they’re the same, then my concern is not warranted, but if they’re not (and R- has higher d’), then this is potentially an issue and would affect the interpretation/results for metacognitive efficiency. I know the authors simulated this situation to try to assess whether the reduction in power would be sufficient to change their results, but I am not convinced that the simulations fully address the issue. In particular, power reductions of “only 0.1-0.3” (line 396) are not marginal! If you only have 0.8 power to start with, a reduction in power of 0.3 is a serious problem! I think this needs to be explored better before alternative explanations can be ruled out.

4. I don’t understand the point of the ML-based discussion. What additional information does this add?

5. The descriptions of some of the simulation procedures are inadequate. Equations would be very helpful.

## Author Response

Dear Dr Postle,

We are grateful to the two reviewers for their detailed reading of our manuscript and constructive comments, and to you for the chance to revise it.

In response to the points raised by the two reviewers, we have now extended the literature review in the Introduction, providing a more thorough account of the relevant literature, old and new. We also report additional analyses of the data that support our interpretations, and added alternative interpretations of our data that we cannot strictly rule out in the Limitations section of the manuscript. Additionally, we have now reworked the entire manuscript to avoid ambiguities in the terms we used to refer to motor contributions to confidence.

We copy below the reviewers’ original comments, along with our responses. We believe that the manuscript has greatly improved in thoroughness and clarity, and hope that it is now suitable for publication in eNeuro.

We thank you again for considering our manuscript for publication.

Sincerely,

The authors of the manuscript [Names edited out for blind review process]

1 Summary

The paper asks the simple question “do overt actions linked to the stimulus improve metacognitive efficiency?” and claims to provide a clear negative answer based on an innovative experimental paradigm where participants perform a confidence judgment on a perceptual decision task, after having tracked the stimulus (CR+) or not (CR-) and emitted a decision (R+) or not (R-).

The paper is as a whole well written and clear. The paradigm is indeed innovative, and the analyses, more generally the strategy, is sound with respect to modern standards (the analytical tools are state of the art, the study is preregistered). Yet I have a few reservations concerning the argumentation, and I think that some improvements in this respect could be helpful:

Response: We first would like to thank Prof. Sackur for his thoughtful, careful and very constructive comments on our manuscript.

1/ The literature reviewed is confined to very recent papers, and while it is obviously good to be up-to-date, one should not miss important older papers. With respect to the link between RTs and confidence judgments, and the locus of confidence computations more generally, I think there are useful ideas and results in Baranski and Petrusic pioneering studies, in Moran et al., 2015, in Pleskac & Busemeyer, 2010 and in Ratcliff & Starns, 2013 for instance, or even, let’s play the archaeologist, Vickers & Packer, 1982 Vickers et al., 1985 - quite relevant on the link between RTs and confidence.

Response: We thank the referee for pointing this out, and for directing us to this very important series of studies. We now included references to them, in the section of the Introduction that we copy below.

"This effect was described very early on (Henmon, 1911; Baranski and Pertusic, 1995), and more recently explained using bounded evidence accumulation models (Plescak and Busemeyer, 2012; Ratcliff and Starns, 2013, Moran et al., 2015). The dependency is strong when accuracy is stressed but is greatly reduced (Vickers and Packer, 1982) or disappears altogether when speed is emphasized, suggesting that the estimation of confidence is flexibly pre- or post-decisional, depending on the task demands (Baranski and Petrusic, 1998). Nevertheless, overall, data from a wide range of recent tasks measuring confidence following discrimination decisions show that an overwhelming majority of participants present a negative relationship between the confidence and decision reaction times (Rahnev et al 2019).”

2/ It seems to me that the discussion of the merits and limits of the paradigm is a bit short. The paradigm is presented as a factorial 2 (continuous tracking of the stimulus (CR): present / absent) X 2 (Overt response (R)): present / absent) design. But in fact, it cannot be analyzed as a factorial design except for one rather peripheral analysis (confidence bias analysis), and the critical analyses are simple comparisons not based on the same response.

Response: The referee is right that this design is only nominally factorial, because the only factorial analysis possible was on mean confidence judgements. We now removed all mentions in the text and figure legends of a factorial design, save for the one in the discussion that indeed refers to the analysis of mean confidence. We opted for modifying the text and not the panel ’Experimental design’ in Figure 1, because we think that it is the clearest way to display the four experimental conditions.

We also now expanded the limitations section, as we describe in our full response to the reviewer’s major remark below.

The real problem lies in the fact that the authors claim that the real import of their proof comes from the part where they use the tracking behavior as a proxy for an unobserved decision. Below I detail why this interpretation of their paradigm is questionable, even beyond the fact that tracking accuracy and decision accuracy do not correlate very well. I think that the critical problem lies in the fact that the very notion of a decision (and the theory of metacognition that goes with it) is tied to the idea that in deciding, participants commit themselves to one categorical option as opposed to another. This is why models of decisions have thresholds or bounds. Tracking is a form of continuous response that does not entail such commitment. In the data that the authors present, I find no evidence of a decision in this categorical sense when there is no overt decision. Of course tracking and decision accuracy correlate (because perceptual sensitivity is involved in both), but this is absolutely no proof of the reality of a decision in the absence of a decisional response. To me, the argument: 1/ remove the empirical evidence for the decision and hope that participants still commit to a decision; 2/ use an objectively non-decisional behavior as a proxy for the hypothesized decision; 3/ apply standard metacognitive reasoning on it, is fragile at best. (As an example of a Bayesian analysis of the subtle link between categorical decisions and continuous responses, see Stocker and Simoncelli, _A Bayesian model of conditioned perception_, 2008.)

Response: We understand that the link between categorical decisions and continuous responses like the one we relied on is subtle, and we thank the referee for pointing us to this reference.

As the reviewer points out below, Stocker and Simoncelli’s paper evaluates the effects of committing to a binary decision on a continuous estimate (at the first level). Along those lines, in fact, Peters et al (Peters, M. a. K., Thesen, T., Ko, Y. D., Maniscalco, B., Carlson, C., Davidson, M., ... Lau, H. (2017). Perceptual confidence neglects decision-incongruent evidence in the brain. Nature Human Behaviour) showed that committing to a decision does in fact affect metacognitive decisions: participants’ confidence judgments tend to ignore evidence against the chosen option in a 2AFC task.

We argue that there is a critical distinction between these two studies and ours: whereas Stocker and Simoncelli and Peters’ studies evaluate the effects of the *identity* of the chosen alternative, we do not compare confidence (or any other continuous measure) for chosen vs unchosen alternatives. In our experiment, by design and by instruction, we compared confidence for decisions that had been committed to, but not expressed behaviourally. We understand this reviewer’s concerns that participants may not have actually committed to a decision. We would like to note however two points that might convince the referee that our approach is not so fragile:

Firstly, we note that we explicitly instructed participants to covertly make a binary decision, even when they did not have to report it. As we discuss in response to a related point below, debriefing with participants after the experiment confirmed they did not adopt other strategies to perform the task.

Secondly, and more importantly, in our experimental design, participants were not informed beforehand whether, on each trial, they would be required to provide a first-order response or not. While this by no means constitutes a proof, it does justify our assumption that participants committed to a decision even in R- trials. We clarified this point in the Limitations section of the revised manuscript.

Some inconsistencies and ambiguities in the vocabulary derive from the above conflations.

Response: As we note below, we have now changed the terms we use to refer to ’continuous report’ (what the reviewer calls tracking behaviour) and ’first-order 2AFC response’ to emphasise the continuous/binary nature of each of the responses.

In all, I think that the paper is very interesting and very well executed as far as the technical parts go, but that it could be strengthened with respect to the precision of the argument.

2 Major remark

══════════════

I have only one major reservation, that I already more or less explained above, and that I will try to expound on now to be clearer, and specify what I think might be another interpretation of the results.

So as to test whether metacognitive accuracy is impacted by the presence of an overt decision, the authors use as a proxy of decision accuracy the tracking behavior (notably ll 313 sqq). But their “proxy” is a valid measure in itself: it is a measure of tracking accuracy (albeit binarized, which may not be necessary). They could have had a confidence judgment on this tracking task. It would have made sense to test whether metacognition for this task is impacted by the presence of a decision - à la Stocker & Simoncelli, 2008, but at the metacognitive level. But now, they compute metacognitive efficiency on a tracking task, based on a confidence judgment that is relative to the decision. So there is some crossover between the tasks at the first and second order. If you give instructions to participants, you should expect that they somehow try to follow them. When you ask them to track the stimulus, you don’t ask them to make a categorical decision; and when you ask them to report the confidence they have in their decision, you don’t ask them how confident they are on their tracking.

It is standard to use proxies, but in doing so, one must realize the alternative and the implications. In fact, due to the above crossover, the situation is completely symmetrical. The decision to take tracking as a proxy for decision is formally on a par with the decision to take decision confidence as a proxy for tracking confidence.

Thus, we could formulate the results the other way around: why don’t call the confidence measure a proxy for the confidence judgment on the tracking task? In that case, the conclusion would be: metacognition efficiency for duration tracking is not impacted by the presence of an overt decision. A conclusion clearly different from “metacognition for decision is not impacted by the presence of first order behavior”. Note that crucially, this other formulation nowhere uses the traditional in the last decade or so, decision related, concept of metacognitive efficiency that is the target of this paper.

Furthermore, I would argue that using confidence in decisions as a proxy for confidence in tracking is more justified than to use tracking as a proxy for decision. Why? Because at the first order level we have incontrovertible behavioral evidence that tracking and decision are opposed on the crucial commitment parameter. We don’t have such strong evidence at the second order level. Ignoramus arguments are weak and one could retort that it may be even worse at the second order level..

Compared to the above analysis, the “second preregistered analysis” (344: CR+R+ / CR-R+) seems completely sound to me.

Such consideration would lead to a reinterpretation of the results: The conclusion drawn from the CR+R+ / CR-R+ comparison would be: “the presence of an overt tracking behavior on the very stimulus on which a decision is based does not impact metacognitive accuracy” and the conclusion drawn from the CR+/R+ / CR+R- comparison would be: “the presence of an overt decision on the very stimulus which is the basis of some tracking behavior does not impact metacognitive accuracy for this tracking”.

With this reinterpretation, the paradigm of the paper would squarely fit in the “third class” of studies reviewed (l. 502), and it would be very far from a “no-report” paradigm.

So I think that the discussion about tracking behavior being a mediocre but still usable proxy for decision misses the point. It is not a matter of quantitative adequacy, but of theoretical distinctiveness. Response times and precision are usually correlated (except when there is a speed-accuracy trade-off), but they are different construct. A DDM clearly explains why we have a correlation, and it would show to what extent one could use one as a proxy for the other.

Response: The reviewer raises one main point from different angles, that we will address at once:

First, as we wrote in our response to one of the comments in the reviewer’s Summary section, we agree that there is a distinction between confidence in a binary decision and confidence in tracking performance. Second, the reviewer notes that we could have focused on comparing confidence in tracking under different conditions, and in particular study the effects that committing to a binary decision has on a continuous confidence judgment. This would represent a complete overhaul of our experimental design, because it would require changing the questions so that a binary decision takes place before a confidence judgement about tracking. We agree with the reviewer that this is an interesting question. But our goal was to inform the many recent studies that investigate confidence reports following binary decisions and, as such, we take our analysis to be the one that is most relevant for the current scientific literature.

But our personal interest is clearly not enough to justify our assumptions and conclusions. Instead, as we argued above, two aspects of our experimental design justify our assumptions. Namely, the task instructions and the fact that participants were not informed during tracking whether they would be asked to commit to a binary decision or not.

While the reviewer points out that it is formally equivalent to take tracking as a proxy for binary decision than the binary decision confidence as a proxy for tracking confidence, we have no reason to believe that participants rated confidence in tracking. Indeed, we examined the data and found no evidence suggesting that participants’ confidence judgments were about different mental representations in R+ vs. R- cases (see figure 1 below).

In the absence of evidence against it, we have no choice but to assume that participants followed the task instructions.

Nevertheless, we agree that it is possible that confidence reports for binary decisions were different than confidence for tracking behaviour, despite our instructions. And it is in principle possible that participants gave two very different kinds of ratings. If this were true, however, we would expect larger, more systematic differences in confidence and/or metacognitive accuracy between CR+R+ and CR+R- conditions. That is, the effects that we investigated would have been artificially easier to detect, because participants would have been doing two very different things in the two different conditions.

This would have been a concern for our results, had we found differences between CR*R+ and CR+R- conditions; because it would have been unclear what was the factor driving these differences. So, this possibility makes it even more striking that we found no differences between these conditions.

Finally, although we do think that our assumptions and analyses are justified, we agree that our interpretations of the data rely heavily on our assumptions being valid. Therefore we now are very clear in pointing out these assumptions at two points in the Discussion section.

- First, at the end of the section under the subheading ’Effect of first-order responses on confidence ratings’:

"We note that in these analyses we assumed that participants had followed our instructions to rate confidence in the binary decision for both committed and omitted responses. We discuss in the Limitations section the implications for our conclusions if participants did not follow these instructions.”

-And also more thoroughly in the Limitations section, where we also describe the reasons why we think that we are justified in considering that the assumptions were met:

"Finally, and importantly, we note that our interpretation of the results relies on two assumptions about trials without overt first-order 2AFC responses: First, we assume that participants committed to a binary decision on CR+R- trials, even though they were not asked to overtly provide one. Second, we assume that participants reported their confidence about the binary decision on both CR+R- and CR+R- trials. In other words, we assume that the only difference between CR+ and CR- conditions, and between R+ and R- conditions, were the manipulations that we induced experimentally (continuous responses and first-order responses, respectively), but that these differences had no impact on the cognitive processes that took place to produce the confidence judgments. If these assumptions were not met, our interpretation would not be valid.

We have reasons to believe that our assumptions are, in fact, justified. First, by instruction: we asked participants to make a decision (and rate their confidence in its accuracy) even in cases where they were not prompted to explicitly provide the answer. Additionally, participants did not know beforehand whether, on each trial, they would have to provide a first-order response. Then, from the participants’ point of view, R+ and R- trial types were indistinguishable until the point of stimulus offset.”

Additionally, following the reviewer’s comment, we now removed the suggestion that our R- conditions constituted an example of a “no-report” paradigm (in the section “Differences with existing literature”).

3 Minor remarks

═══════════════

3.1 Abstract

────────────

The fact that “across a broad range of tasks, trials with quick reaction times to the first-order task are often judged with relatively higher confidence than those with slow responses” is of course no proof that “confidence could be informed by a readout of reaction times in addition to decision-monitoring processes", but it is not even really suggestive of that, as it is quite obvious that most models of decision will have parameters that impact both response times and confidence, without the later having to be in any way based on the former (see Vickers, 1985; Pleskac & Busemeyer, 2010; Ratcliff & Starns, 2013).

Response: We agree with the referee that a negative correlation between confidence and first-order reaction times is in many models accounted for, without the need to invoke a causal mechanism. We now refer to more direct evidence in favor of a link between the two:

"In fact, some recent studies have revealed that directly manipulating motor regions in the brain, or the time of first-order decisions relative to second-order ones affects confidence judgements.”

I find the last sentence of the abstract a bit unclear. I don’t understand why the rejection of the null hypothesis (as the preview of the results has it) would have rejected the notion that the relationship between first order and confidence judgments is “correlational”.

Response: We simplified the last sentence of the abstract, also in line with the change that was suggested in the comment above this one. It now reads:

"These results suggest that confidence ratings do not always incorporate motor information.”

3.2 Significance statement

──────────────────────────

I would remove the reference to the brain. In metacognition, it’s the mind that monitors itself. The brain might be said to monitor itself when someone is looking at his / her own real time fMRI or EEG.

Response: We agree that, given that we report no electrophysiological data in this paper, it might be misleading to talk about the brain. We now define metacognition as the “ability to monitor one’s own thoughts”.

The assertion that only post-decisional signals is “truly metacognitive” is a bit rash. I think it conflates retrospection with metacognition. A judgment might be authentically metacognitive (second order) while being made as the decision unfolds. Cf Fleming & Daw, 2017.

Response: We fully agree, and have changed the significance statement accordingly:

"In this paradigm, both first- and second-order information could, in principle, influence confidence judgements.”

3.3 Introduction

────────────────

l. 44 reference to “perceptual judgment” is too narrow, as it also applies to other tasks, as the authors state themselves on ll. 52 sq.

Response: It is true that metacognitive tasks have moved beyond perceptual and memory judgements to include the monitoring of, for example, attention or motor control. But the overwhelming majority of studies still focus on perceptual and memory decision making, We think that it is much clearer to focus on a narrow, but still representative kind of study in the field. We modified the sentence, it now reads:

"In what has become a standard paradigm, participants first make a binary decision (typically, a perceptual or memory judgement, first-order task) and immediately afterwards give a measure of confidence in their response (second-order task).”

l. 77 The sentence “A drift-diffusion model explained this effect by showing that the accumulation of perceptual evidence is constrained by first-order decisions.” is unclear. First, for a reader not acquainted with Pereira et al., 2018, it is unclear how one could provide a DDM for an observed decision. Is it a DDM of the confidence judgment RT? Otherwise I don’t really understand how “perceptual evidence accumulation” could not be related to first order decision? In the sequential sampling framework isn’t perceptual evidence accumulation the crucial process of first order decisions?

Response: The DDM in the paper by Pereira et al is not standard (i.e., fitted to observed decisions) and therefore difficult to explain briefly in the introduction. It is not crucial in this paper, so we removed this sentence and simply state that “metacognitive performance for decisions that are committed with a key press is better than that to equivalent decisions that are observed (Pereira et al., 2018)”.

3.4 Material and methods

────────────────────────

The psychophysical description, while probably not very important, is incomplete. For instance, what is a “maximum luminance” “sinusoidal grating”? What is the speed of the drift?

Response: We now describe the stimuli more clearly:

"Stimuli were red or green vertical gratings that drifted sideways. The gratings were formed by a sine-wave function (0.27 cycles/{degree sign}), drifting sideways at 15{degree sign}/s, and drawn inside a square (8{degree sign} height and width), presented at fixation. The green and red stimuli always drifted left- and rightwards respectively.”

By “maximum luminance” we meant an alpha value of one. This was unclear, so we now report [RGBA] values for each color instead.

It might be useful at some early point to specify that the task is a duration comparison.

Response: We added the following sentence, before describing the four different conditions:

"The task consisted of a duration comparison, followed by a confidence rating.”

Similarly, it would be very useful to learn whether the design was randomized or blocked very early. In fact, it is nowhere stated, but we need to know either how participants are cued for each conditions if it is a randomized design and block duration and counterbalancing if it is a blocked design.

Response: We thank the referee for pointing out that this important piece of information was missing. We now clarify at the end of the description of the task that:

"Trial types were interleaved, and the order of the trials was randomized for each participant. On any given trial, participants were not informed beforehand whether a first-order response would be required. That is, after stimulus offset, participants were either prompted to give a 2AFC on the colour corresponding to the longest duration, or were directly prompted to give a confidence rating.”

l. 149 I have a real methodological worry concerning R- trials (trials with “covert decision”). The authors state: “In conditions without first-order response (R-), participants were also required to make a temporal summation decision (the decision was overt in R+ trials but covert in R- trials).” But how can we be sure that participants do as they are asked? For all we know participants might not do anything in this case and use the confidence scale to report their confidence in their tracking performance; or use the confidence scale as a means for reporting their first order response... With so little knowledge about whether participants are or are not committing to a decision in R- blocks, even a a qualitative debriefing would be useful.

Response: Ensuring that participants do as they are asked is indeed crucial in experimental psychology in general, especially in cases where we ask for subjective reports. We verified that participants followed instructions in several ways. First, experimenters always checked participants’ behavior during the training phase, and reminded them that when no response was to be provided, they still had to make a binary decision covertly, “in their own mind”. Second, experimenters debriefed all participants at the end of the experiment, and verified informally the strategies put in place to perform the task. No participant reported using the confidence scale to report confidence in tracking or first-order response. Third, beyond qualitative evidence, we also inspected confidence distributions visually to confirm that they were similar between conditions, suggesting that participants rated confidence about the same quantity, and not about a binary (CR+R+) vs. continuous decision (CR+R-) as suggested by the referee. We include the plots below this response. We also examined individual confidence distributions (not shown here). And, even in single-participant confidence distributions, we again found that the pattern was consistent within individual and across the different conditions.

Finally we note that, had we found differences in metacognitive sensitivity between conditions with and without first-order response (R+/R-), it would have been crucial to control for, for example, the kind of task-engagement effects that the referee suggests. The fact that we found no differences in metacognitive measures between the conditions suggest that even these potential differences in behaviour did not have a significant effect on our metacognitive measures.

Figure 1: Confidence distributions between experimental conditions.

As for the use of Fleming’s hierarchical Bayesian toolbox for the estimation of meta-d’/d’, it would be useful to have some details concerning the estimation procedure: number of chains, of samples, length of adaptation, starting values (all that for the MCMC procedure). Then we would need some information about the quality of the estimation (at least Gelman-Rubin tests). Most importantly we need to know the hierarchical structure when comparing conditions (eg, l 361, do the authors do a separate hierarchical estimate for each condition, or do they use a regression, with only one estimate?) Most of that could be in SMs.

Response: The referee is right that these technical details may be relevant for some readers, and have added them in the Methods section of the manuscript, along with convergence checks.

For the MCMC procedure, we used 4 chains of 10000 iterations including 1000 for adaptation, no thinning, and default initial values as generated by JAGS. Separate hierarchical estimates were computed for each condition. Potential scale reduction factors regarding average M-ratio estimates were equal to 1.02 (CR+R+), 1.02 (CR-R+), 1.06 (CR+R+) and 1.16 (CR+R-). Only the last value for CR+R- indicates a possible lack of convergence, so we refitted the model with 30000 iterations including 10000 for warmup, which resulted in scale reduction factors of 1.03 and 1.11, respectively, with no difference in M-ratios between conditions. These values still point to possible converge problems, presumably due to the relatively low number of trials in our sample. We take these results with caution and base our discussion on the (otherwise consistent) results from the logistic regression analyses.

3.5 Analyses

────────────

I don’t quite understand the interim conclusion l. 281: “Together, these results indicate that fast first-order responses were associated with higher confidence, but that response times are unlikely to play a causal role as removing first-order responses altogether had no effect on confidence.” As I understand it the last analysis before this sentence includes RTs, so how could it prove anything about the causal role of RTs?

I think that the authors oscillate between treating the continuous response as a first order response (they need that when there is no other first order behavior) or an accompaniment of the first order decision (they need that so that they can test its causal impact). This is fine, but here they seem to imply that while their model always include RTs, they can consider condition (CR+/CR-) as the difference between RT present and absent...

Response: Our interim conclusion may have been unclear, as we meant to summarize the results from the two first analyses together. We rephrased it and it now reads:

"Together, the results of these two analyses on mean confidence and the relationship between first-order RT and confidence indicate that fast first-order responses were associated with higher confidence, but that response times are unlikely to play a causal role as removing first-order responses altogether had no effect on mean confidence.”

In other words, the observation that ’removing first-order responses altogether had no effect on confidence’ does stem from the absence of a main effect of R+ (and not CR+) in the factorial analysis on mean confidence. Therefore, we do not make, as the referee suggests, any assumption on RTs by contrasting CR+ and CR- trials.

l. 299 Perhaps some ambiguities could be resolved by using the phrase “first order behavior” for the continuous tracking response. When I read the sentence: “We therefore expected conditions with overt first-order responses to be associated with better metacognitive sensitivity (measured as the relationship between confidence and first-order accuracy) than those without motor responses” my first impulse is to find that incoherent as there cannot be any first order accuracy when there is no first order response. And the problem is sometimes even worse when the first order tracking behavior is used as a proxy for the absent first order response.

Response: We agree with the reviewer that our choice of naming was confusing. We now use ’tracking behavior’ and ’first-order 2-alternative forced choice’ (or ’first-order 2AFC’) throughout the manuscript.

ll 301sqq The factorial design seems neat, but still, the authors cannot eat their cake and have it: in case when there is no first order decision and when they use the tracking behavior as a proxy for the decision, they cannot still compare the effect of tracking present vs absent on metacognition efficiency. Unless they use some magical trick that I don’t understand. In other words, What is the use of the tracking absent / first order decision absent in the estimation of metacognitive efficiency?

Response: We originally included the CR-R- condition with the aim to fully assess the contribution of both kinds of motor behaviour on confidence. But the referee is right that the design is not really factorial, as only the pairwise comparisons CR+R+ vs. CR+R- and CR+R+ vs. CR-R+ are meaningful when comparing measures of metacognition. We re-wrote this paragraph so that we no longer refer to “conditions without first-order responses” and instead refer specifically, in line with our response to the previous comment, to ’trials without responses to the 2AFC first-order task’. We also added the following sentence:

"We then compared metacognitive sensitivity between conditions with and without first-order responses. It is only possible to estimate metacognitive sensitivity in R- trials if they are also CR+. In other words, we required the continuous report from CR+ conditions to estimate metacognitive sensitivity in cases of no first-order response (R-). Therefore, we built a mixed-effects logistic regression for proxy accuracy that included condition (CR+R+/CR+R-) and confidence and their interaction as fixed effects, ...”

The transition on line 363 is a bit surprising, because it comes just after the CR+R+ / CR-R+ analysis that is not based on any proxy. The wording of the transition to this part could be better formulated. More generally, I find the presentation of these simulations not optimal. It was already presented in abstracto at the beginning of the Analysis section, but it is only here that one can really understand their justification. And it does not seem crucial to the argument. I would put the whole of it in an appendix / supplementary materials section.

Response: We thank the referee for this suggestion. We now provide a more detailed description of our model with equations, and removed the short presentation at the beginning of the analysis section which may have been misleading. We would like to keep these simulations in the main text, as they are crucial to support our behavioral results. We are willing to revise this judgment upon editorial request.

Similarly, the very short ML paragraph would comfortably sit in a supplementary material section.

Response: We agree with the reviewer but, unfortunately, eNeuro does not support Supplementary material. Given the option to delete it completely or include it in the main text, and in the spirit of open science, we decided to include it in the main text, as we did already. Here again, we are willing to revise this decision upon editorial request.

3.6 Discussion

──────────────

I am not really convinced by the explanation of the increased in absolute mean confidence in the CR+ condition compared to the CR-, based on “attentional demands”: why increasing attention should increase confidence? Do we have some previous literature on that? If anything, Rahnev et al., 2013 find the opposite. In a sense, I might expect that all things equal, increased attention might increase metacognitive efficiency. But why should it make participants overconfident?

Response: The reviewer is right that studies like that of Rahnev et al. 2011 showed that increasing attention decreases confidence, and this is, in fact, an argument against, and not for, simple attentional factors explaining our results.

We now instead focus on the result and point out that:

"Over conditions with and without first-order responses (both R+ and R-), we found a consistent increase in confidence following continuous report (CR+ vs. CR-) despite no changes in first-order performance. Previous studies have shown that different factors can affect first- and second-order performance independently. These factors include experimental manipulations like changes in stimulus variability (Spence et al, 2015) or sensory reliability (Bang and Fleming, 2018), pharmacological silencing of different brain regions (Stolyarova et al, 2019), as well as the existence of sub-threshold motor activity (Gajdos et al, 2019), differences in movement parameters (Faivre et al., 2019) or voluntary control (Charles et al, 2019). Our study adds a novel kind of manipulation, namely the occurrence of motor responses, to the list of experimental manipulations that affect confidence but not first-order accuracy.”

.

l. 524 Reyes & Sackur, 2014 revolves around the notion that introspection is flexible and tries to makes the best of whatever information is available (perceptual, decisional, motor).

Response: We thank the referee for pointing us to this relevant and interesting paper. At the end of the paragraph beginning on l. 524 (in the original manuscript), we now added a reference to it.

"This admittedly speculative account is in line with a previous study (Reyes and Sackur, 2014) showing that introspective report in a visual search task (that is, subjective reports about the number of items scanned, or the time required to scan them) may rely on different sources of information depending on the task context.”

l. 546 shouldn’t it be “overt” instead of “covert”?

Response: This was unclear, and we changed the sentence which now reads:

"While voluntary key presses paired to the stimuli shown on the screen were a relatively poor predictor of first-order responses, ...”

4 Very minor remarks

════════════════════

Legend of Figure 2. What is the difference between “mean confidence” and “confidence” on the y-axis of panel A and B. I think that “raw RT” would be clearer than “continuous RT” (all the more than there is a continuous duration task in some conditions...)

Response: We labelled the y-axes differently because in one case, statistics were done on the mean confidence judgments for each participant (figure 2.A); whereas in the other case, the mixed-linear models operate at the trial level so no average confidence per participant is considered (figure 2.B). We explained this by stating that “For illustrative purposes, we plot open circles and error bars that represent mean {plus minus} 95%CI over participants after rounding reaction times and subtracting 0.5 s”. We now clarify this further by indicating in the figure legend that the ANOVA was done on mean confidence, whereas the linear mixed-effects regressions represent trial-wise analyses.

In Vuillaume et al., 2019 in some conditions participants observe the behaviors and have access to the stimulus, contrary to what is implied on line 500.

Response: We now clarify that it was only on some conditions that participants were asked to judge confidence based on observed reaction times.

I always sign my reviews

Jérôme Sackur

Response: Once again, we would like to thank Prof. Sackur for this very thoughtful and thorough review.

-

Reviewer #2

The authors hypothesize that a readout of reaction time could lead to confidence judgments, in addition to monitoring of internal decisional processes. I’m a big proponent of trying to uncover what exactly goes into confidence judgments, so I am glad to see this type of work being done. However, my enthusiasm is somewhat dampened here due to some issues which I describe below.

Primarily, I am a little concerned about the implied novelty of this manuscript. Only one citation of Kiani et al., 2014 exists, but it does not appear to inform the formation of the present hypotheses. This is interesting because Kiani et al.’s paper is literally titled “Choice certainty is informed by both evidence and decision time” -- seemingly a direct precursor to the present manuscript’s goals. Additionally, no citations exist of Fetsch et al., 2014 or Zylberberg et al., 2016, both of which use this “accumulation time” model to successfully explain seemingly counterintuitive findings in confidence judgments. In fact, the latter (Zylberberg et al.) explicitly investigates how stimulus volatility and reaction time together lead to the construction of confidence.

Response: We are sorry to read that the reviewer is not convinced of the novelty of our results. As he/she suggests, one aspect of our paradigm was indeed to quantify confidence ratings in the presence and absence of first-order response. When first-order responses are removed, confidence ratings should change; and their accuracy, measured as metacognitive sensitivity, should decrease. We agree that we should have given more credit to the important studies mentioned by the reviewer testing a similar hypothesis. We now include references to the three studies (Kiani et al., 2014; Fetsch et al., 2014, Zylberberg et al., 2016) that the reviewer suggested.

We note, however, that these studies have a rather different approach than ours. In all cases, the perceptual evidence was experimentally manipulated. Kiani et al. used a random-dot motion task, and showed that transiently presenting participants with evidence against the correct choice extended decision times and decreased confidence judgments without affecting first-order accuracy. Fetsch and colleagues (2014) added perceptual noise directly to the brain, through electrical microstimulation in rhesus monkeys. And Zylberberg et al. (2016) mimicked this effect in human volunteers by adding ’volatility’ to moving dots displayed (whilst also confirming the effect of the experimental manipulation through electrophysiological recordings in monkeys). They found that noisier evidence accumulation processes led to decreases in reaction times and increases in confidence. As the reviewer points out, in all cases these effects could be accounted for by bounded accumulator models that provide common grounds for choice, reaction time and evidence. Critically, the approach we take in our experiment is not to alter the perceptual evidence presented in any way, but to remove the sensorimotor evidence paired to it.

In these papers, the argument is often put forward that these experimental manipulations reveal a dissociation between first- and second-order judgments because the changes in confidence are statistically detectable, whereas those in the first-order judgement are not. But the fact that differences in first-order performance are not statistically detectable does not mean that they are not there. Indeed, perceptual evidence feeds directly into the first-order judgment. In our experiment, instead, we do manipulate only the information that, we hypothesized, fed into the second-order (but not first-order) judgements.

We now clarify this in the manuscript:

"Experimental manipulations that artificially change the process of evidence accumulation have provided strong mechanistic explanations for this relationship (Kiani et al., 2014; Fetsch et al., 2014, Zylberberg et al., 2016). But these manipulations ultimately affected the evidence available to the observer, or the process of accumulation itself. Here, we sought to compare confidence judgements and metacognitive performance between conditions that differed only on the sensorimotor information available for the decision, but that were indistinguishable from the point of view of perceptual evidence.”

We argue in the Discussion that that through our (indeed) novel manipulation of first-order responses, we unveiled a flexible aspect of the computation of confidence:

"We speculate that the computation of confidence may be flexible, and largely depend on the information that is globally available. In all previous studies, to the best of our knowledge, participants had access to some form of first-order reaction time information, at some point in time during the trial: either through observation from the third-person perspective, directly after the confidence report or through simple access to reaction times produced under experimentally manipulated motor signals. In some conditions of our experiment, instead, responses were completely absent and may have shifted participant’s global strategies for the computation of confidence. In other words, we contest that while first-order reaction time information is, under some experimental settings, used by participants to generate a confidence judgement, when motor information is not available at all, it may be replaced by other, equally precise sources of information, closer to the strength of evidence...”

In addition to the inadequate literature review, I have a few theoretical concerns about the project.

1. The staircase procedures used throughout the block mean that metacognitive sensitivity will be over-estimated (Rahnev & Fleming, 2019). While the authors acknowledge this possibility, they do not investigate how staircase variability changed from one block to the next or how such variability could have impacted metacognitive sensitivity estimates.

Response: Rahnev and Fleming showed that it was the variability in the stimulus difficulty that leads to inflation of estimates of metacognitive ability. Then, this might be a problem in within-subject comparisons if two conditions differ in the range (or standard deviation) of the stimulus strengths presented. In two separate analyses, we checked whether this was the case in our data by comparing the range and standard deviation of the stimuli presented to each participant in the CR+ and CR- conditions (we remind the reviewer that the staircases for R+ and R- conditions were yoked to their corresponding condition based on CR). In both cases, likelihood ratio tests revealed strong evidence for the null hypothesis:

Range of stimulus strengths presented:

χ2 = 0.1075, p = 0.743, difference in Bayesian Information Criterion = -8.56, BF10 = 0.014

Standard deviation of stimulus strengths presented:

χ2 = 0.01, p = 0.752, difference in Bayesian Information Criterion = -10.86, BF10 = 0.004

We added these results in a new short section under the subtitle Effects of experimental manipulation.

2. Relatedly, this is especially important given that confidence judgments have been shown to depend on absolute as well as relative evidence favoring a decision (e.g. Zylberberg et al., 2012; Maniscalco et al., 2016; Samaha et al., 2016; Peters et al., 2017; Odegaard et al., 2018). “Absolute evidence” has been defined differentially as contrast or % of coherently moving dots, among other variants. This means that the continuous staircase changes are not only affecting meta-d’/d’, but also potentially confidence judgment magnitudes themselves in ways unaccounted for by the analyses or signal detection theoretic power simulations. It is unclear how the authors may deal with this, as “perceptual evidence” e.g. line 278) appears to refer to relative and not absolute evidence.

Response: If we understand the reviewer correctly, point 2 is only relevant if there are differences between conditions in the variability of the stimuli presented, and we have shown in our response to this reviewer’s point 1 that this is not the case.

Additionally, we note that our arguments are based on comparisons between conditions where, in both cases, the same definition of perceptual evidence applies.

3. I am concerned about the assumption that “the percept associated with longer key-presses during continuous report corresponded to the covert first-order response” (line 303-304). In the simulation, the authors state that the correspondence between overt and covert responses was about 70%. This suggests that at low evidence levels, the effect of noise is considerable. This is confirmed by the fact that “response predictability based on the stimulus ... and proxy ... was significantly higher than based on the stimulus alone” (lines 307-309). I worry that this lack of correspondence may unduly inflate meta-d’/d’ judgments, because the participant’s d’ as defined via this proxy measure may be higher than the d’ actually experienced by the subject. That the staircase showed matched performance between R+ and R- trials does not fix this: if proxy-defined d’ in R- = reported d’ in R+, but *true* d’ is actually lower in R-, then d’ in R- will seem artificially inflated, thus reducing the meta-d’/d’ ratio because the too-high d’ is in the denominator. The authors could test this by comparing proxy-defined d’ in R+ and R- conditions -- Figure 3a seems to suggest there may be a small but significant effect. If they’re the same, then my concern is not warranted, but if they’re not (and R- has higher d’), then this is potentially an issue and would affect the interpretation/results for metacognitive efficiency. I know the authors simulated this situation to try to assess whether the reduction in power would be sufficient to change their results, but I am not convinced that the simulations fully address the issue. In particular, power reductions of “only 0.1-0.3” (line 396) are not marginal! If you only have 0.8 power to start with, a reduction in power of 0.3 is a serious problem! I think this needs to be explored better before alternative explanations can be ruled out.

Response: As we understand it, the reviewer raises several points here.

Comment: “This is confirmed by the fact that “response predictability based on the stimulus ... and proxy ... was significantly higher than based on the stimulus alone” (lines 307-309).”

Response: There might be a misunderstanding here. The result described in lines 307-309 in fact argues that, while noisy, the proxy does carry meaningful information. Because we staircased the first-order task difficulty, all participants had approximately 71% correct responses. In other words, the stimulus shown is already a very good predictor of behaviour, with 71% accuracy. The fact that a model including both stimulus and proxy explained more variance in the responses than the stimuli alone shows that the noise in the proxy is “low enough", because it adds predictive power to an already rather good predictor of behaviour. In response to this comment, we now added the following clarification of this result in the manuscript, as follows:

"In other words, the proxy, derived from the motor tracking behaviour, consistently added predictive power to the stimulus presented, which was an already good guess (i.e., above-chance, ∼71%) for the covert response provided in the first-order task.”

Comment: “I worry that this lack of correspondence may unduly inflate meta-d’/d’ judgments, because the participant’s d’ as defined via this proxy measure may be higher than the d’ actually experienced by the subject.”

Response: Here there might be another misunderstanding. Precisely to avoid possible systematic biases in our data, we always compared proxy responses between conditions, and never proxy responses vs. overt responses where (as the reviewer points out) biases in d’ would be plausible. We think that this point alone should be enough to address the reviewer’s concerns regarding our comparisons between R+ and R- conditions.

But the following points further argue for the validity of our analyses:

i. Unlike what happens for second-order responses (where both theoretical accounts and empirical data show that injecting perceptual noise leads to higher confidence ratings, Rahnev et al, 2011), injecting noise before the first-order decision (in this case, when obtaining the proxy) is not expected to lead to consistently higher d’. So there is no a-priori reason why this should be the case. (Still, we ran this analysis, see below)

ii. Our conclusions are not based on metad’/d’ measures alone, they are also confirmed by logistic regression analyses, where d’ is not in the denominator.

iii. The reviewer notes that the fact that “the staircase showed matched performance between R+ and R- trials does not fix this”. In fact, we show matched performance between *CR+ and CR-* trials. We did not do the online staircase in R- trials so that we did not have to commit to a specific proxy for behaviour during stimulus presentation (that is, before being able to analyze the data and affecting participants’ behaviour by altering the trial difficulty). Still, to properly address this point, we ran the d’ comparisons suggested by the reviewer.

iv. We agree that the simulations do not specifically address potential concerns about differences in d’ between conditions. The best way to address it is, as the reviewer suggests, to compare proxy-based d’ values between for R+ and R- trials. That is, CR+R+ vs CR+R- trials. estimate d’ for all participants. We found no differences in the d’ of proxy-based responses between conditions with and without first-order responses, and instead evidence in favour of the null hypothesis of no difference between them (χ2 =0.696, p = 0.404, difference in Bayesian Information Criterion = -5.887, BF10 = 0.053)

We also now clarify that the reductions in power in the range of 0.1-0.3 “may partially, but not completely, explain our results"

4. I don’t understand the point of the ML-based discussion. What additional information does this add?

Response: If the analysis had yielded a result (namely, if ML methods would have helped us get a better predictor of covert responses), it would have been an interesting result to report, and we would have built on that to produce a better proxy for covert decisions. We deem null findings to be important, and this is why we decided to include them in the main text originally.

Reviewer 1 suggested that we include this information as Supplementary Information instead, and we think that this would have been the best compromise. However, as we point out in our response above, eNeuro does not support Supplementary material. For that reason, we have kept this paragraph in the main text but are willing to revise this decision on editorial request.

5. The descriptions of some of the simulation procedures are inadequate. Equations would be very helpful.

Response: The complication of the simulations lies in its algorithm, not the equations themselves. So we think that the best way to detail the simulations is by providing the code that we used to run them, which we did already. But, as per suggestion of the reviewer, we have now added the relevant equations in the section in the Methods where we describe the simulations.
