# Supplementary material for: Response-Related Signals Increase Confidence But Not Metacognitive Performance
Source: eNeuro. 2020 May 20;7(3):ENEURO.0326-19.2020. doi: 10.1523/ENEURO.0326-19.2020 (PMC7240286; doi:10.1523/ENEURO.0326-19.2020)
Supplement: Extended Data — Supplementary Experimental codes, raw data, analysis, and simulation files. Download Extended Data, ZIP file. [file enu-eN-NWR-0326-19-s01.zip › filevich_metareport_revised/analysis/analysis_master.html]

Response-related signals increase confidence but not metacognitive performance


# Response-related signals increase confidence but not metacognitive performance

# Prepare data

## Check which subjects should be excluded

## Exclude Subjects

## Discretize (Re-scale and bin) confidence

# Run LMERs

## Choose your confidence measure of interest

```
rawData$confChosen <- rawData$confScaled
#rawData$confChosen <- rawData$conf
```

## Get subsets of data with relevant trials

```
data.withRT1 <- rawData[rawData$type1=="R1+",]
data.withoutRT1 <- rawData[rawData$type1=="R1-",]
data.withContRep <- rawData[rawData$contReport =="CR+",]
data.withRT1_CR <- rawData[(rawData$contReport =="CR+" & rawData$type1=="R1+"),] 
data.withRT1_noCR <- rawData[(rawData$contReport =="CR-" & rawData$type1=="R1+"),]
```

# Sanity checks/Describe data

### Mean(SD) percent correct

```
## 
##  Paired t-test
## 
## data:  meanCorrectType1 by contReport
## t = 0.1196, df = 22, p-value = 0.9059
## alternative hypothesis: true difference in means is not equal to 0
## 95 percent confidence interval:
##  -0.01774111  0.01991257
## sample estimates:
## mean of the differences 
##             0.001085727
```

```
## 
## Cohen's d
## 
## d estimate: 0.0245045 (negligible)
## 95 percent confidence interval:
##     lower     upper 
## -0.388486  0.437495
```

```
## Warning: data coerced from tibble to data frame
```

```
## Bayes factor analysis
## --------------
## [1] Alt., r=0.707 : 0.2930947 ±0.02%
## 
## Against denominator:
##   Null, mu1-mu2 = 0 
## ---
## Bayes factor type: BFindepSample, JZS
```

### Mean(SD) duration difference (converged staircase value)

```
## 
##  Paired t-test
## 
## data:  meanStaircaseValue by contReport
## t = 0.48036, df = 22, p-value = 0.6357
## alternative hypothesis: true difference in means is not equal to 0
## 95 percent confidence interval:
##  -0.04871602  0.07808656
## sample estimates:
## mean of the differences 
##              0.01468527
```

```
## 
## Cohen's d
## 
## d estimate: 0.09187935 (negligible)
## 95 percent confidence interval:
##      lower      upper 
## -0.2944171  0.4781758
```

```
## Warning: data coerced from tibble to data frame
```

```
## Bayes factor analysis
## --------------
## [1] Alt., r=0.707 : 0.3055773 ±0.02%
## 
## Against denominator:
##   Null, mu1-mu2 = 0 
## ---
## Bayes factor type: BFindepSample, JZS
```

### Accuracy of the proxy

```
##          suj meanProxyCorrect
## 1      041AR        0.7192982
## 2      060CG        0.5833333
## 3      110KA        0.6530612
## 4      180IG        0.6842105
## 5      191LG        0.6140351
## 6      200MR        0.5689655
## 7      230MT        0.7818182
## 8      301ER        0.7000000
## 9  PF_CH_001        0.5172414
## 10 PF_CH_002        0.7288136
## 11 PF_CH_003        0.6206897
## 12 PF_CH_005        0.5555556
## 13 PF_CH_007        0.7966102
## 14 PF_CH_008        0.5932203
## 15 PF_CH_009        0.7586207
## 16 PF_CH_010        0.7200000
## 17 PF_CH_011        0.6909091
## 18 PF_CH_012        0.5000000
## 19 PF_CH_013        0.6440678
## 20 PF_CH_014        0.6400000
## 21 PF_CH_015        0.6166667
## 22 PF_CH_016        0.6949153
## 23 PF_CH_017        0.6842105
```

```
## [1] 0.5
```

```
## [1] 0.7966102
```

```
## [1] 0.655054
```

```
## [1] 0.08011776
```

### RT1 predicts confidence?

```
##  Family: gaussian 
##   Links: mu = identity; sigma = identity 
## Formula: confChosen ~ type1RT * combiCondition + durationDifference + (type1RT + combiCondition | suj) 
##    Data: data.withRT1 (Number of observations: 2559) 
## Samples: 4 chains, each with iter = 2000; warmup = 1000; thin = 1;
##          total post-warmup samples = 4000
## 
## Group-Level Effects: 
## ~suj (Number of levels: 23) 
##                                      Estimate Est.Error l-95% CI u-95% CI
## sd(Intercept)                           12.02      2.13     8.51    16.90
## sd(type1RT)                              6.35      1.42     4.03     9.69
## sd(combiConditionCRP_R1P)                5.67      1.66     2.59     9.12
## cor(Intercept,type1RT)                  -0.17      0.24    -0.60     0.33
## cor(Intercept,combiConditionCRP_R1P)    -0.27      0.25    -0.70     0.29
## cor(type1RT,combiConditionCRP_R1P)       0.26      0.28    -0.32     0.77
##                                      Eff.Sample Rhat
## sd(Intercept)                              1733 1.00
## sd(type1RT)                                1438 1.00
## sd(combiConditionCRP_R1P)                  1109 1.00
## cor(Intercept,type1RT)                     1557 1.00
## cor(Intercept,combiConditionCRP_R1P)       2536 1.00
## cor(type1RT,combiConditionCRP_R1P)         2097 1.00
## 
## Population-Level Effects: 
##                               Estimate Est.Error l-95% CI u-95% CI
## Intercept                        66.97      2.93    61.31    72.93
## type1RT                         -15.17      1.65   -18.48   -11.98
## combiConditionCRP_R1P             1.54      1.86    -2.21     5.19
## durationDifference               25.23      4.19    17.18    33.29
## type1RT:combiConditionCRP_R1P     0.86      1.24    -1.61     3.26
##                               Eff.Sample Rhat
## Intercept                           1443 1.00
## type1RT                             1924 1.00
## combiConditionCRP_R1P               2905 1.00
## durationDifference                  4413 1.00
## type1RT:combiConditionCRP_R1P       4555 1.00
## 
## Family Specific Parameters: 
##       Estimate Est.Error l-95% CI u-95% CI Eff.Sample Rhat
## sigma    21.70      0.31    21.10    22.31       8004 1.00
## 
## Samples were drawn using sampling(NUTS). For each parameter, Eff.Sample 
## is a crude measure of effective sample size, and Rhat is the potential 
## scale reduction factor on split chains (at convergence, Rhat = 1).
```

```
## Hypothesis Tests for class b:
##      Hypothesis Estimate Est.Error CI.Lower CI.Upper Evid.Ratio Post.Prob
## 1     main conf   -15.17      1.65   -17.95   -12.53        Inf      1.00
## 2     main cond     1.54      1.86    -1.54     4.54       4.04      0.80
## 3 main evidence    25.23      4.19    18.40    31.98        Inf      1.00
## 4     conf*cond     0.86      1.24    -1.20     2.90       3.19      0.76
##   Star
## 1    *
## 2     
## 3    *
## 4     
## ---
## 'CI': 90%-CI for one-sided and 95%-CI for two-sided hypotheses.
## '*': For one-sided hypotheses, the posterior probability exceeds 95%;
## for two-sided hypotheses, the value tested against lies outside the 95%-CI.
## Posterior probabilities of point hypotheses assume equal prior probabilities.
```

```
## Note: uncertainty of error terms are not taken into account. You may want to use `rstantools::posterior_predict()`.
```

Yes, as we expected, type1RT clearly predicts confidence (in the subset of trials with type1)

### Interaction RT1\*accuracy predicts confidence?

```
##  Family: gaussian 
##   Links: mu = identity; sigma = identity 
## Formula: conf ~ type1RT * contReport * correctFactor + durationDifference + (type1RT + contReport + correctFactor + durationDifference | suj) 
##    Data: data.withRT1 (Number of observations: 2559) 
## Samples: 4 chains, each with iter = 2000; warmup = 1000; thin = 1;
##          total post-warmup samples = 4000
## 
## Group-Level Effects: 
## ~suj (Number of levels: 23) 
##                                                Estimate Est.Error l-95% CI
## sd(Intercept)                                      0.15      0.03     0.10
## sd(type1RT)                                        0.05      0.01     0.03
## sd(contReportCRP)                                  0.05      0.02     0.02
## sd(correctFactorIncorrect)                         0.05      0.02     0.02
## sd(durationDifference)                             0.16      0.06     0.05
## cor(Intercept,type1RT)                            -0.16      0.24    -0.60
## cor(Intercept,contReportCRP)                      -0.26      0.25    -0.68
## cor(type1RT,contReportCRP)                         0.28      0.28    -0.31
## cor(Intercept,correctFactorIncorrect)              0.21      0.26    -0.31
## cor(type1RT,correctFactorIncorrect)                0.17      0.27    -0.38
## cor(contReportCRP,correctFactorIncorrect)         -0.21      0.32    -0.76
## cor(Intercept,durationDifference)                 -0.55      0.25    -0.90
## cor(type1RT,durationDifference)                    0.04      0.32    -0.59
## cor(contReportCRP,durationDifference)              0.06      0.36    -0.64
## cor(correctFactorIncorrect,durationDifference)    -0.08      0.32    -0.67
##                                                u-95% CI Eff.Sample Rhat
## sd(Intercept)                                      0.21       1508 1.00
## sd(type1RT)                                        0.08       1794 1.00
## sd(contReportCRP)                                  0.08       1155 1.00
## sd(correctFactorIncorrect)                         0.09       1999 1.00
## sd(durationDifference)                             0.29       1158 1.00
## cor(Intercept,type1RT)                             0.33       1715 1.00
## cor(Intercept,contReportCRP)                       0.25       2797 1.00
## cor(type1RT,contReportCRP)                         0.75       2072 1.00
## cor(Intercept,correctFactorIncorrect)              0.70       3250 1.00
## cor(type1RT,correctFactorIncorrect)                0.69       2788 1.00
## cor(contReportCRP,correctFactorIncorrect)          0.46       1871 1.00
## cor(Intercept,durationDifference)                  0.06       1632 1.00
## cor(type1RT,durationDifference)                    0.66       2110 1.00
## cor(contReportCRP,durationDifference)              0.71       2190 1.00
## cor(correctFactorIncorrect,durationDifference)     0.54       2180 1.00
## 
## Population-Level Effects: 
##                                              Estimate Est.Error l-95% CI
## Intercept                                        0.71      0.04     0.64
## type1RT                                         -0.15      0.02    -0.18
## contReportCRP                                    0.01      0.02    -0.03
## correctFactorIncorrect                          -0.15      0.03    -0.20
## durationDifference                               0.22      0.06     0.11
## type1RT:contReportCRP                           -0.00      0.01    -0.03
## type1RT:correctFactorIncorrect                   0.04      0.02     0.00
## contReportCRP:correctFactorIncorrect            -0.00      0.03    -0.06
## type1RT:contReportCRP:correctFactorIncorrect     0.01      0.02    -0.03
##                                              u-95% CI Eff.Sample Rhat
## Intercept                                        0.78        875 1.01
## type1RT                                         -0.12       1537 1.00
## contReportCRP                                    0.05       2385 1.00
## correctFactorIncorrect                          -0.10       2089 1.00
## durationDifference                               0.34       1985 1.00
## type1RT:contReportCRP                            0.03       2624 1.00
## type1RT:correctFactorIncorrect                   0.07       2206 1.00
## contReportCRP:correctFactorIncorrect             0.06       2221 1.00
## type1RT:contReportCRP:correctFactorIncorrect     0.06       1958 1.00
## 
## Family Specific Parameters: 
##       Estimate Est.Error l-95% CI u-95% CI Eff.Sample Rhat
## sigma     0.21      0.00     0.20     0.22       6390 1.00
## 
## Samples were drawn using sampling(NUTS). For each parameter, Eff.Sample 
## is a crude measure of effective sample size, and Rhat is the potential 
## scale reduction factor on split chains (at convergence, Rhat = 1).
```

```
## Hypothesis Tests for class b:
##      Hypothesis Estimate Est.Error CI.Lower CI.Upper Evid.Ratio Post.Prob
## 1       main RT    -0.15      0.02    -0.17    -0.12        Inf      1.00
## 2     main cond     0.01      0.02    -0.02     0.04       0.41      0.29
## 3      main cor    -0.15      0.03    -0.19    -0.10        Inf      1.00
## 4 main evidence     0.22      0.06     0.12     0.32        Inf      1.00
## 5     conf*cond     0.00      0.01    -0.03     0.02       1.06      0.52
## 6      conf*cor     0.04      0.02     0.01     0.07      46.06      0.98
## 7 conf*cond*cor     0.01      0.02    -0.03     0.05       2.85      0.74
##   Star
## 1    *
## 2     
## 3    *
## 4    *
## 5     
## 6    *
## 7     
## ---
## 'CI': 90%-CI for one-sided and 95%-CI for two-sided hypotheses.
## '*': For one-sided hypotheses, the posterior probability exceeds 95%;
## for two-sided hypotheses, the value tested against lies outside the 95%-CI.
## Posterior probabilities of point hypotheses assume equal prior probabilities.
```

```
## Note: uncertainty of error terms are not taken into account. You may want to use `rstantools::posterior_predict()`.
```

```
## Warning: Removed 35 rows containing non-finite values (stat_summary).
```

```
## Warning: Removed 2 rows containing missing values (geom_path).
```

### Mean confidence between conditions

1. Does Type1 as a factor (present/absent) affect confidence?
2. Does ContReport as a factor (present/absent) affect confidence?

```
##  Family: gaussian 
##   Links: mu = identity; sigma = identity 
## Formula: conf ~ type1 * contReport + (type1 + contReport | suj) 
##    Data: rawData (Number of observations: 5243) 
## Samples: 4 chains, each with iter = 2000; warmup = 1000; thin = 1;
##          total post-warmup samples = 4000
## 
## Group-Level Effects: 
## ~suj (Number of levels: 23) 
##                              Estimate Est.Error l-95% CI u-95% CI
## sd(Intercept)                    0.13      0.02     0.10     0.19
## sd(type1R1P)                     0.06      0.01     0.04     0.09
## sd(contReportCRP)                0.06      0.01     0.04     0.09
## cor(Intercept,type1R1P)         -0.12      0.23    -0.55     0.34
## cor(Intercept,contReportCRP)    -0.42      0.20    -0.74     0.02
## cor(type1R1P,contReportCRP)      0.01      0.25    -0.48     0.50
##                              Eff.Sample Rhat
## sd(Intercept)                      1061 1.00
## sd(type1R1P)                       1552 1.00
## sd(contReportCRP)                  1188 1.00
## cor(Intercept,type1R1P)            2051 1.00
## cor(Intercept,contReportCRP)       2363 1.00
## cor(type1R1P,contReportCRP)        1496 1.00
## 
## Population-Level Effects: 
##                        Estimate Est.Error l-95% CI u-95% CI Eff.Sample
## Intercept                  0.58      0.03     0.52     0.64        661
## type1R1P                   0.01      0.02    -0.02     0.04       1949
## contReportCRP              0.04      0.02     0.01     0.07       1536
## type1R1P:contReportCRP    -0.02      0.01    -0.04     0.01       5150
##                        Rhat
## Intercept              1.00
## type1R1P               1.00
## contReportCRP          1.00
## type1R1P:contReportCRP 1.00
## 
## Family Specific Parameters: 
##       Estimate Est.Error l-95% CI u-95% CI Eff.Sample Rhat
## sigma     0.24      0.00     0.24     0.24       6763 1.00
## 
## Samples were drawn using sampling(NUTS). For each parameter, Eff.Sample 
## is a crude measure of effective sample size, and Rhat is the potential 
## scale reduction factor on split chains (at convergence, Rhat = 1).
```

```
## Hypothesis Tests for class b:
##        Hypothesis Estimate Est.Error CI.Lower CI.Upper Evid.Ratio
## 1      main type1     0.01      0.02    -0.01     0.04       3.43
## 2 main contreport     0.04      0.02     0.01     0.06      75.92
## 3      main inter    -0.02      0.01    -0.04     0.00       0.10
##   Post.Prob Star
## 1      0.77     
## 2      0.99    *
## 3      0.09     
## ---
## 'CI': 90%-CI for one-sided and 95%-CI for two-sided hypotheses.
## '*': For one-sided hypotheses, the posterior probability exceeds 95%;
## for two-sided hypotheses, the value tested against lies outside the 95%-CI.
## Posterior probabilities of point hypotheses assume equal prior probabilities.
```

```
##  Family: gaussian 
##   Links: mu = identity; sigma = identity 
## Formula: conf ~ type1 * contReport + (type1 + contReport | suj) 
##    Data: rawData (Number of observations: 5243) 
## Samples: 4 chains, each with iter = 2000; warmup = 1000; thin = 1;
##          total post-warmup samples = 4000
## 
## Group-Level Effects: 
## ~suj (Number of levels: 23) 
##                              Estimate Est.Error l-95% CI u-95% CI
## sd(Intercept)                    0.13      0.02     0.10     0.19
## sd(type1R1P)                     0.06      0.01     0.04     0.09
## sd(contReportCRP)                0.06      0.01     0.04     0.09
## cor(Intercept,type1R1P)         -0.12      0.23    -0.55     0.34
## cor(Intercept,contReportCRP)    -0.42      0.20    -0.74     0.02
## cor(type1R1P,contReportCRP)      0.01      0.25    -0.48     0.50
##                              Eff.Sample Rhat
## sd(Intercept)                      1061 1.00
## sd(type1R1P)                       1552 1.00
## sd(contReportCRP)                  1188 1.00
## cor(Intercept,type1R1P)            2051 1.00
## cor(Intercept,contReportCRP)       2363 1.00
## cor(type1R1P,contReportCRP)        1496 1.00
## 
## Population-Level Effects: 
##                        Estimate Est.Error l-95% CI u-95% CI Eff.Sample
## Intercept                  0.58      0.03     0.52     0.64        661
## type1R1P                   0.01      0.02    -0.02     0.04       1949
## contReportCRP              0.04      0.02     0.01     0.07       1536
## type1R1P:contReportCRP    -0.02      0.01    -0.04     0.01       5150
##                        Rhat
## Intercept              1.00
## type1R1P               1.00
## contReportCRP          1.00
## type1R1P:contReportCRP 1.00
## 
## Family Specific Parameters: 
##       Estimate Est.Error l-95% CI u-95% CI Eff.Sample Rhat
## sigma     0.24      0.00     0.24     0.24       6763 1.00
## 
## Samples were drawn using sampling(NUTS). For each parameter, Eff.Sample 
## is a crude measure of effective sample size, and Rhat is the potential 
## scale reduction factor on split chains (at convergence, Rhat = 1).
```

```
## `stat_bindot()` using `bins = 30`. Pick better value with `binwidth`.
```

No. responding to the type1 question alone does **NOT** add confidence. Yet providing a continuous report increases confidence.

# Confirmatory analyses

### Does type1 as a factor affect metacognitive ability?

We need some proxy for correct/incorrect. we will use continuous report and the estimated proxies

```
##  Family: bernoulli 
##   Links: mu = logit 
## Formula: proxyIsLongerPercept ~ conf * combiCondition + (conf + combiCondition | suj) 
##    Data: data.withContRep (Number of observations: 2578) 
## Samples: 4 chains, each with iter = 2000; warmup = 1000; thin = 1;
##          total post-warmup samples = 4000
## 
## Group-Level Effects: 
## ~suj (Number of levels: 23) 
##                                      Estimate Est.Error l-95% CI u-95% CI
## sd(Intercept)                            0.89      0.22     0.54     1.40
## sd(conf)                                 0.53      0.34     0.04     1.31
## sd(combiConditionCRP_R1P)                0.19      0.13     0.01     0.51
## cor(Intercept,conf)                     -0.32      0.43    -0.90     0.69
## cor(Intercept,combiConditionCRP_R1P)    -0.04      0.45    -0.84     0.83
## cor(conf,combiConditionCRP_R1P)          0.03      0.49    -0.85     0.87
##                                      Eff.Sample Rhat
## sd(Intercept)                              1232 1.00
## sd(conf)                                    533 1.00
## sd(combiConditionCRP_R1P)                  1537 1.00
## cor(Intercept,conf)                        1536 1.00
## cor(Intercept,combiConditionCRP_R1P)       4257 1.00
## cor(conf,combiConditionCRP_R1P)            1972 1.00
## 
## Population-Level Effects: 
##                            Estimate Est.Error l-95% CI u-95% CI Eff.Sample
## Intercept                      0.95      0.28     0.41     1.53       1422
## conf                           0.82      0.34     0.13     1.48       2327
## combiConditionCRP_R1P          0.00      0.26    -0.48     0.53       2281
## conf:combiConditionCRP_R1P    -0.11      0.39    -0.89     0.66       2343
##                            Rhat
## Intercept                  1.00
## conf                       1.00
## combiConditionCRP_R1P      1.00
## conf:combiConditionCRP_R1P 1.00
## 
## Samples were drawn using sampling(NUTS). For each parameter, Eff.Sample 
## is a crude measure of effective sample size, and Rhat is the potential 
## scale reduction factor on split chains (at convergence, Rhat = 1).
```

```
## Hypothesis Tests for class b:
##   Hypothesis Estimate Est.Error CI.Lower CI.Upper Evid.Ratio Post.Prob
## 1  main conf     0.82      0.34     0.26     1.36     116.65      0.99
## 2  main cond     0.00      0.26    -0.41     0.43       0.99      0.50
## 3  conf*cond    -0.11      0.39    -0.76     0.52       1.57      0.61
##   Star
## 1    *
## 2     
## 3     
## ---
## 'CI': 90%-CI for one-sided and 95%-CI for two-sided hypotheses.
## '*': For one-sided hypotheses, the posterior probability exceeds 95%;
## for two-sided hypotheses, the value tested against lies outside the 95%-CI.
## Posterior probabilities of point hypotheses assume equal prior probabilities.
```

```
## Note: uncertainty of error terms are not taken into account. You may want to use `rstantools::posterior_predict()`.
```

```
## Scale for 'colour' is already present. Adding another scale for
## 'colour', which will replace the existing scale.
```

```
## Scale for 'fill' is already present. Adding another scale for 'fill',
## which will replace the existing scale.
```

```
## Scale for 'y' is already present. Adding another scale for 'y', which
## will replace the existing scale.
```

There’s no interaction between type1 and the effect of confidence - so no differences on the metacognitve side. So we find **no differences** in metacognitive ability and **no differences** in confidence following type1 as a factor.

Now let’s look at continuous report. We saw earlier that continuous Report (as a factor) affects confidence. So:

### Does adding CR on top of stimulus categoy help to predict responses?

```
m1 = glmer(factor(resp1) ~ stim + (1  | suj), data = data.withRT1[data.withRT1$combiCondition=='CR+_R1+',], family = binomial)
m2 = glmer(factor(resp1) ~ stim +predictedAnswer + (1  | suj), data = data.withRT1[data.withRT1$combiCondition=='CR+_R1+',], family = binomial)
anova(m1,m2)
```

```
## Data: data.withRT1[data.withRT1$combiCondition == "CR+_R1+", ]
## Models:
## m1: factor(resp1) ~ stim + (1 | suj)
## m2: factor(resp1) ~ stim + predictedAnswer + (1 | suj)
##    Df    AIC    BIC  logLik deviance  Chisq Chi Df Pr(>Chisq)   
## m1  3 1525.8 1541.3 -759.91   1519.8                            
## m2  4 1517.8 1538.4 -754.89   1509.8 10.038      1   0.001533 **
## ---
## Signif. codes:  0 '***' 0.001 '**' 0.01 '*' 0.05 '.' 0.1 ' ' 1
```

### Does CR+ lead to higher metacognitive ability?

```
##  Family: bernoulli 
##   Links: mu = logit 
## Formula: correct ~ conf * combiCondition + (conf + combiCondition | suj) 
##    Data: data.withRT1 (Number of observations: 2620) 
## Samples: 4 chains, each with iter = 2000; warmup = 1000; thin = 1;
##          total post-warmup samples = 4000
## 
## Group-Level Effects: 
## ~suj (Number of levels: 23) 
##                                      Estimate Est.Error l-95% CI u-95% CI
## sd(Intercept)                            0.68      0.22     0.31     1.17
## sd(conf)                                 1.25      0.32     0.70     1.95
## sd(combiConditionCRP_R1P)                0.10      0.08     0.00     0.30
## cor(Intercept,conf)                     -0.85      0.13    -0.98    -0.51
## cor(Intercept,combiConditionCRP_R1P)     0.01      0.48    -0.84     0.88
## cor(conf,combiConditionCRP_R1P)         -0.10      0.48    -0.91     0.83
##                                      Eff.Sample Rhat
## sd(Intercept)                              1229 1.00
## sd(conf)                                   1157 1.00
## sd(combiConditionCRP_R1P)                  3067 1.00
## cor(Intercept,conf)                         907 1.00
## cor(Intercept,combiConditionCRP_R1P)       6417 1.00
## cor(conf,combiConditionCRP_R1P)            6202 1.00
## 
## Population-Level Effects: 
##                            Estimate Est.Error l-95% CI u-95% CI Eff.Sample
## Intercept                     -0.38      0.22    -0.81     0.06       2391
## conf                           2.51      0.39     1.75     3.29       2260
## combiConditionCRP_R1P         -0.16      0.22    -0.58     0.28       3678
## conf:combiConditionCRP_R1P     0.13      0.36    -0.60     0.84       3655
##                            Rhat
## Intercept                  1.00
## conf                       1.00
## combiConditionCRP_R1P      1.00
## conf:combiConditionCRP_R1P 1.00
## 
## Samples were drawn using sampling(NUTS). For each parameter, Eff.Sample 
## is a crude measure of effective sample size, and Rhat is the potential 
## scale reduction factor on split chains (at convergence, Rhat = 1).
```

```
## Hypothesis Tests for class b:
##   Hypothesis Estimate Est.Error CI.Lower CI.Upper Evid.Ratio Post.Prob
## 1  main conf     2.51      0.39     1.88     3.16        Inf      1.00
## 2  main cond    -0.16      0.22    -0.52     0.20       0.30      0.23
## 3  conf*cond     0.13      0.36    -0.47     0.73       1.77      0.64
##   Star
## 1    *
## 2     
## 3     
## ---
## 'CI': 90%-CI for one-sided and 95%-CI for two-sided hypotheses.
## '*': For one-sided hypotheses, the posterior probability exceeds 95%;
## for two-sided hypotheses, the value tested against lies outside the 95%-CI.
## Posterior probabilities of point hypotheses assume equal prior probabilities.
```

```
## Note: uncertainty of error terms are not taken into account. You may want to use `rstantools::posterior_predict()`.
```

```
## Scale for 'colour' is already present. Adding another scale for
## 'colour', which will replace the existing scale.
```

```
## Scale for 'fill' is already present. Adding another scale for 'fill',
## which will replace the existing scale.
```

```
## Scale for 'y' is already present. Adding another scale for 'y', which
## will replace the existing scale.
```

No interaction between contReport and the effect of confidence. So no differences on the metacognitve side

### Bayesian verion

```
## 
## Attaching package: 'magrittr'
```

```
## The following object is masked from 'package:purrr':
## 
##     set_names
```

```
## The following object is masked from 'package:tidyr':
## 
##     extract
```

```
## 
## Attaching package: 'reshape2'
```

```
## The following object is masked from 'package:tidyr':
## 
##     smiths
```

```
## Linked to JAGS 4.3.0
```

```
## Loaded modules: basemod,bugs
```

```
## 
## Attaching package: 'ggpubr'
```

```
## The following object is masked from 'package:plyr':
## 
##     mutate
```

```
## 
## Attaching package: 'ggmcmc'
```

```
## The following object is masked from 'package:bayestestR':
## 
##     ci
```

# Generate complete figures

### Figure 2: Effects on confidence (descriptive analyses)

```
## `stat_bindot()` using `bins = 30`. Pick better value with `binwidth`.
```

```
## `stat_bindot()` using `bins = 30`. Pick better value with `binwidth`.
```

```
## png 
##   2
```

### Figure 3: Effects on metacognitive sensitivity (confirmatory analyses)

```
## png 
##   2
```
