# Supplementary figures and images for: Response-Related Signals Increase Confidence But Not Metacognitive Performance
Source: eNeuro. 2020 May 20;7(3):ENEURO.0326-19.2020. doi: 10.1523/ENEURO.0326-19.2020 (PMC7240286; doi:10.1523/ENEURO.0326-19.2020)

A.

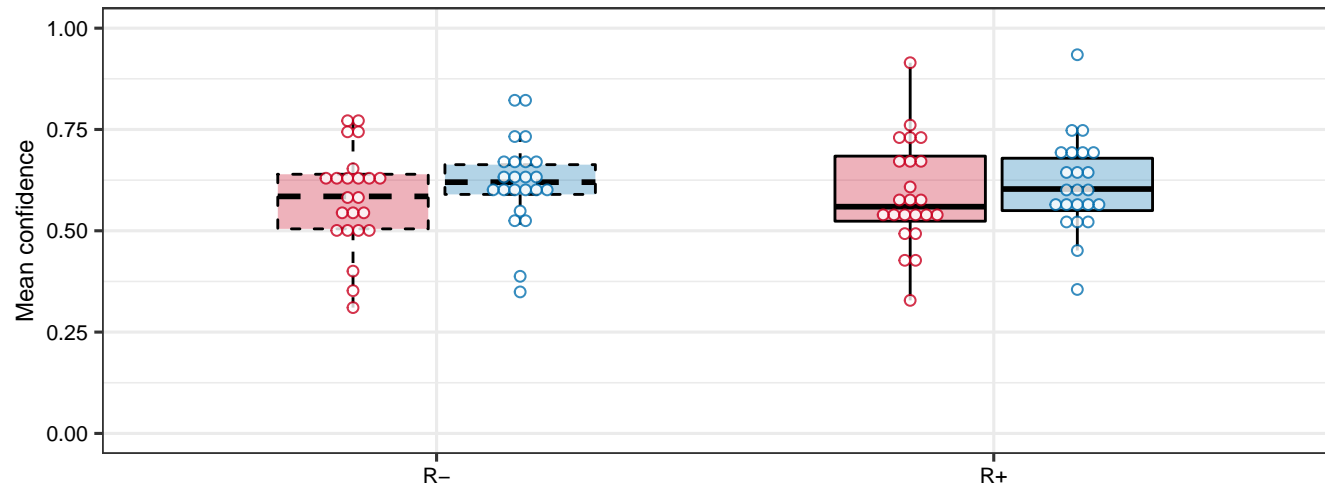

B.

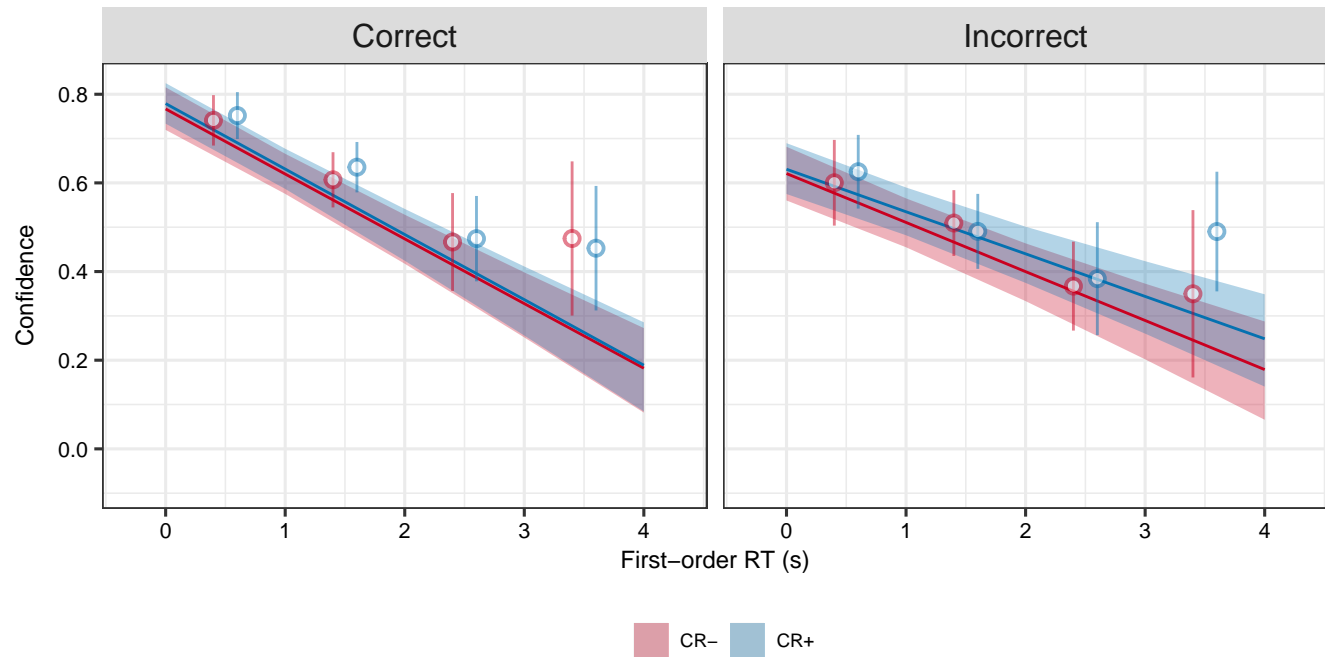

Supplement: Extended Data — Supplementary Experimental codes, raw data, analysis, and simulation files. Download Extended Data, ZIP file. [file enu-eN-NWR-0326-19-s01.zip › filevich_metareport_revised/analysis/figures/Figure2.pdf]

A.

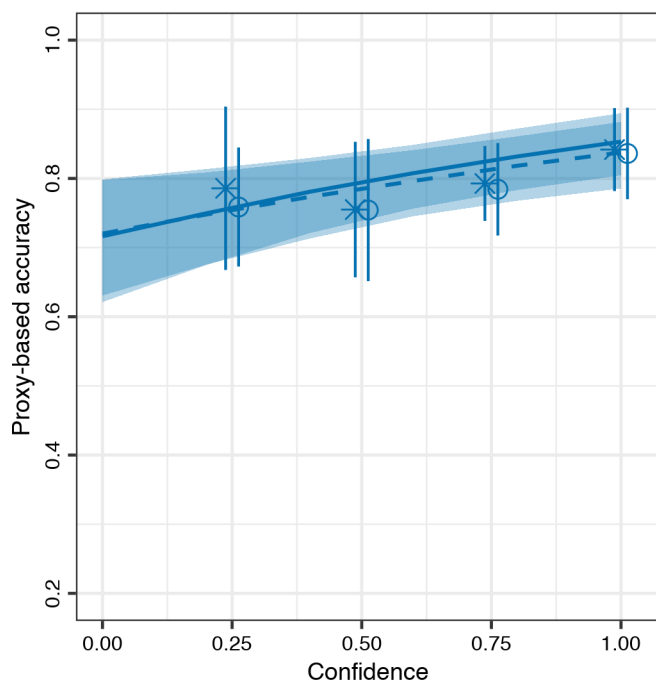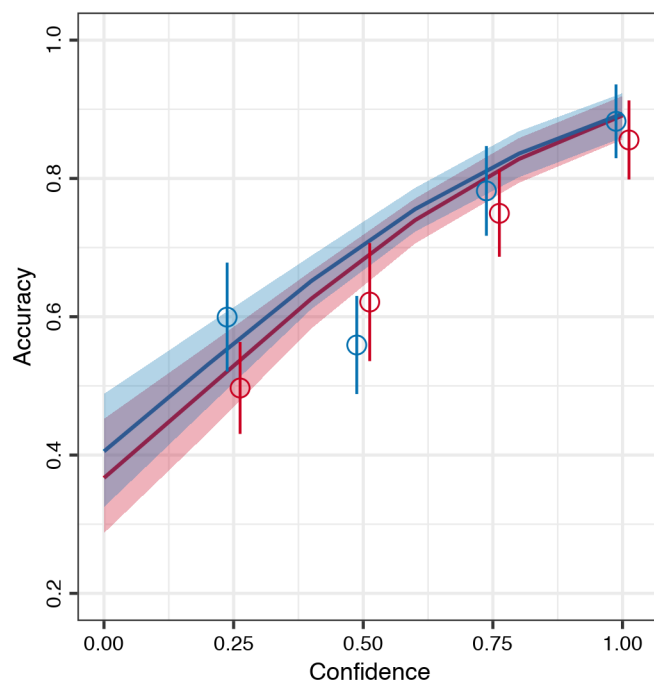

B.

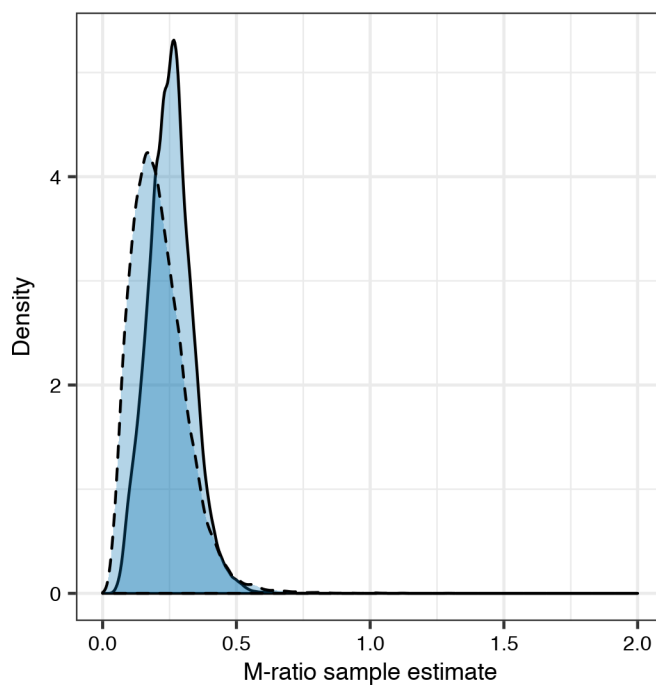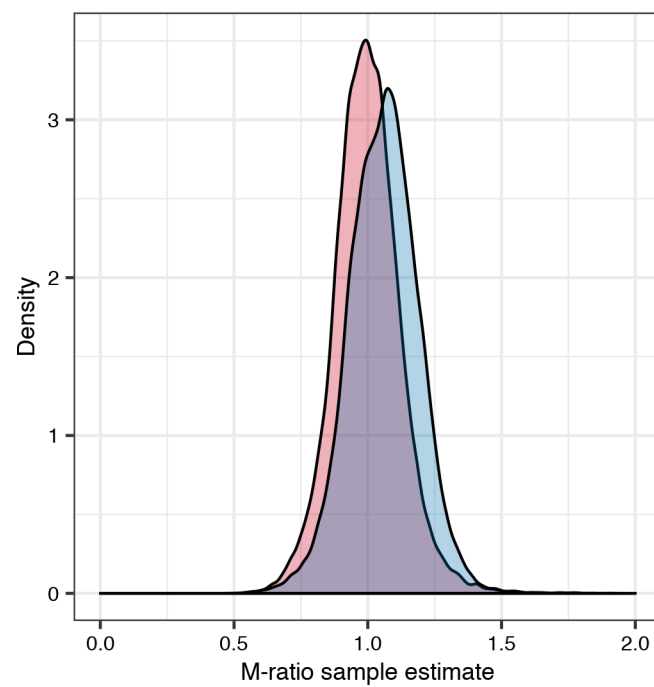

Supplement: Extended Data — Supplementary Experimental codes, raw data, analysis, and simulation files. Download Extended Data, ZIP file. [file enu-eN-NWR-0326-19-s01.zip › filevich_metareport_revised/analysis/figures/Figure3.pdf]
